# The Role of Natural Hydrogels in Enhancing Wound Healing: From Biomaterials to Bioactive Therapies

**DOI:** 10.3390/pharmaceutics17101243

**Published:** 2025-09-23

**Authors:** Paula Stefana Pintilei, Roya Binaymotlagh, Laura Chronopoulou, Cleofe Palocci

**Affiliations:** 1 Department of Chemistry, Sapienza University of Rome, Piazzale Aldo Moro 5, 00185 Rome, Italy; paulastefana.pintilei@uniroma1.it (P.S.P.); roya.binaymotlagh@uniroma1.it (R.B.); laura.chronopoulou@uniroma1.it (L.C.); 2 Research Center for Applied Sciences to the Safeguard of Environment and Cultural Heritage (CIABC), Sapienza University of Rome, Piazzale Aldo Moro 5, 00185 Rome, Italy

**Keywords:** wound healing, natural hydrogels, tissue regeneration, biocompatibility, biodegradability, controlled drug release, antimicrobial hydrogels, nanoparticles

## Abstract

Wound healing is a complex, multifaceted biological process that plays a vital role in recovery and overall quality of life. However, conventional wound care methods often prove insufficient, resulting in delayed healing, higher infection risk, and other complications. In response, biomaterials—especially hydrogels—have gained attention for their advanced wound management capabilities, which support wound healing by maintaining moisture, mimicking the extracellular matrix (ECM), and enabling targeted drug delivery triggered by wound-specific signals. They frequently carry antimicrobial or anti-inflammatory agents, promote blood vessel and nerve regeneration, and are biocompatible with customizable properties suited to different healing stages. Natural hydrogels, derived from polysaccharides, proteins, and peptides, offer several advantages over synthetic options, including inherent bioactivity, enzymatic degradability, and cell-adhesive qualities that closely resemble the native ECM. These features facilitate cell interaction, modulate inflammation, and speed up tissue remodeling. Moreover, natural hydrogels can be engineered as delivery systems for therapeutic agents like antimicrobial compounds, nanoparticles, growth factors, and exosomes. This review discusses recent advances in the use of natural hydrogels as multifunctional wound dressings and delivery platforms, with a focus on their composition, mechanisms of action, and potential for treating chronic and infected wounds by incorporating antimicrobial and regenerative additives such as silver and zinc oxide nanoparticles.

## 1. Introduction

### 1.1. Skin

Human skin is a remarkable organ that serves as the body’s first barrier of defense against external threats. As the largest organ of the integumentary system, the skin makes up approximately 15% of total body weight and drapes an average surface area of about 1.5 to 2.0 square meters in adults [1,2]. It protects against injuries, germs, chemicals, and UV radiation while helping to control body temperature, allowing us to feel sensations, and even supporting vitamin D production [3]. Because of this wide, multifarious array of protective and regulatory functions, skin integrity is vital to human health, and any disruption in its structure or function can lead to a cascade of local and systemic complications [4]. Several factors, including nutrition, hydration, and cell regeneration, contribute to the skin’s ability to repair itself after injury [5]. The biochemical balance of skin lipids and proteins plays a crucial role in maintaining its function, highlighting the importance of external influences such as climate and skincare products [6].

Structurally, the skin is traditionally divided into three main layers: the epidermis, the dermis, and the hypodermis (also called subcutaneous tissue). Each layer contains specific cellular and extracellular elements that contribute to particular functional properties. Together, these three layers form a highly adaptive and robust organ system that can respond dynamically to a wide range of environmental and internal stimuli [7]. Understanding the structural organization and functional interaction among the epidermis, dermis, and hypodermis is critical to the development of research in dermatology, tissue engineering, wound healing, and transdermal drug delivery. Regenerative medicine has come a long way in skin repair methods, developing bioengineered skin grafts and new wound healing strategies [8]. Transdermal drug delivery systems have been improved with innovative techniques, which enhance topical treatment efficacy through controlled drug absorption of therapeutic agents [9]. Nanotechnology also optimized drug penetration across the skin barrier, with enhanced treatment efficacy for various dermatological conditions [10]. Skin is not just a passive barrier but an active one in terms of immune defense and homeostasis maintenance because, as the first line of defense, it is exposed to the outer environment [11]. Its cellular heterogeneity, multilayered structure, and physiological flexibility highlight its essential importance in both health and disease [12].

The skin also has several protective functions. Its physical barrier keeps out harmful microorganisms and shields against injuries [13]. Sweat and oil produced by glands create a slightly acidic surface, preventing bacteria and fungi from growing, while antimicrobial peptides such as defensins neutralize potential threats [14]. Specialized immune cells, including Langerhans cells, help recognize dangerous invaders and trigger immune responses [15]. Sensory receptors embedded in the skin detect heat, cold, pressure, pain, and vibrations, allowing the body to react to environmental changes [16]. Lastly, melanocytes produce melanin, which absorbs UV radiation and helps protect against DNA damage and skin cancer [17].

### 1.2. Wound Healing

Wound healing is a carefully regulated biological process that occurs in four overlapping phases: hemostasis, inflammation, proliferation, and maturation. The process begins immediately after injury with hemostasis, where blood vessels constrict and platelets form a clot to stop bleeding. This clot also releases chemical signals that trigger the healing cascade. Soon after, the inflammation phase begins. White blood cells—mainly neutrophils and then macrophages—arrive at the site to remove pathogens, damaged cells, and debris, while also releasing growth factors to guide the next steps [18,19]. Once the wound is cleared, the proliferative phase takes over. During this stage, fibroblasts produce collagen and extracellular matrix components, creating granulation tissue that supports new cell growth. Simultaneously, keratinocytes move across the wound surface to regenerate the epidermis, and new capillaries form to restore blood flow and supply nutrients [20,21]. The final stage is maturation or remodeling. Here, collagen is realigned, excess blood vessels regress, and the new tissue gains strength and flexibility. This phase can be slow, lasting from weeks to several months—or even longer—depending on the wound and the individual’s health [22,23]. Various factors like diabetes, infection, poor circulation, or a weakened immune system can interfere with this sequence, often causing wounds to stall in a specific phase and become chronic [24,25]. Table 1 shows the most important stages in the speed and quality of wound healing.

Wound healing is influenced by a variety of internal and external factors that can either support or impair the process (Table 2). Positive influences include proper wound care—such as cleaning, dressing, and infection control—adequate nutrition rich in protein, vitamins A and C, and zinc, as well as maintaining moisture balance, which promotes faster epithelialization. Patient education also plays a critical role in improving adherence to care routines and overall healing outcomes. Conversely, negative factors like infection, poor circulation (especially in conditions such as diabetes or vascular disease), smoking, chronic illnesses like obesity and autoimmune disorders, and psychological stress or sleep deprivation can significantly delay healing [26]. Clinically, effective monitoring of wound healing involves assessing wound size and depth, the type of tissue present (e.g., granulation or necrotic), the nature of exudate, the condition of wound edges and surrounding skin, and pain levels. These parameters help determine healing progression and whether additional intervention is necessary [27].

### 1.3. Acute and Chronic Wounds

Wounds result from injured skin or underlying tissues caused by several etiologies, including physical trauma, burns, chemical injury, or infections. They are generally categorized into two general categories: acute wounds, which resolve in a predictable, timely manner, and chronic wounds, which fail to heal properly and persist for extended periods. This is important to determine treatment and the course of recovery [28]. Acute wounds follow a predictable healing process that includes four main stages: hemostasis, inflammation, proliferation, and remodeling [29]. Acute wounds typically heal within a few days to several weeks, depending on the severity and the depth of the injury [30]. Common examples include cuts, abrasions, puncture wounds, burns, and surgical wounds [31]. These wounds generally heal without complications if properly managed, but can be caused by infection, poor circulation, and systemic illness [32].

Chronic wounds, on the other hand, fail to heal within the expected timeframe and often persist for months or years [33]. Chronic wounds are commonly associated with systemic conditions such as diabetes, vascular diseases, long-term immobility, and autoimmune disorders [34]. Chronicity of the wound is caused by disruption of normal healing of the wound by chronic inflammation, excessive production of matrix metalloproteinases (MMPs), aberrant angiogenesis, and reduced oxygen supply [35]. Additionally, biofilms of bacteria tend to form on chronic wounds, which make the wounds antibiotic and immune cell-resistant [36]. Some examples of chronic wounds include diabetic foot ulcers, which occur as a result of nerve damage and circulatory insufficiency in diabetic patients [37]; venous leg ulcers, caused by poor blood flow in the veins, leading to tissue destruction [38]; pressure ulcers, found in immobile patients due to prolonged pressure on the skin [39]; and arterial ulcers, which occur due to severe arterial deficiency, leading to tissue necrosis [40]. Table 3 demonstrates the fundamental differences between acute and chronic wounds.

Treating wounds presents several challenges, particularly when dealing with chronic wounds, which tend to heal much more slowly than acute ones. Infection is a common complication, as open wounds are vulnerable to bacterial invasion [41]. In many cases, these bacteria form biofilms—protective layers that shield them from antibiotics and the body’s immune defenses, making infections harder to eliminate [42,43]. Excessive inflammation is another issue; while inflammation is a necessary part of healing, if it becomes too severe or prolonged, it can damage healthy tissue and delay the healing process. Additionally, during the remodeling phase, disorganized or excessive collagen production can lead to fibrotic scarring [44], which may be both esthetically undesirable and functionally limiting, particularly when it affects joint mobility. Individual factors such as age, nutrition, overall health, and lifestyle habits like smoking also significantly influence the speed and quality of wound healing [45,46]. Understanding the differences between acute and chronic wounds is essential for developing effective treatment strategies and improving patient outcomes [47]. While acute wounds usually heal without complications, chronic wounds often require targeted, specialized care to address underlying systemic issues and prevent long-term consequences [48]. Ongoing research into the biology of wound healing and the development of advanced therapies continues to enhance treatment protocols and improve recovery outcomes for patients with complex or non-healing wounds [49].

## 2. Conventional Clinical Practices in Wound Healing

Wound care relies on a variety of conventional treatment methods designed to support healing by protecting the wound, controlling infection, removing damaged tissue, and improving blood flow [50,51]. These methods range from simple dressings to more involved procedures. For example, dry gauze is widely used because it is affordable and accessible, but it tends to dry out the wound and can stick to new tissue, causing discomfort during dressing changes [52,53]. In contrast, moist dressings like hydrocolloids and foam dressings create a better environment for healing by maintaining moisture and encouraging tissue growth, though they may not always prevent infection or disrupt bacterial biofilms effectively [54]. Topical antimicrobials such as iodine or silver are also applied to reduce bacterial contamination, but their effects can be short-lived, and they sometimes cause irritation to healthy tissue [55]. Removing dead tissue is another key step in wound management, achieved through different types of debridement [56]. Surgical removal is fast but invasive, while mechanical methods like wet-to-dry dressings can be painful and less selective. Enzymatic and autolytic debridement are gentler alternatives that encourage the body’s own healing processes but tend to work more slowly [57]. For more complex wounds, negative pressure wound therapy (NPWT) has become a popular option, using suction to clear excess fluid and promote new tissue formation, though it requires specialized equipment and training [58]. Compression therapy is a mainstay in treating venous ulcers by reducing swelling and improving circulation, but it must be used cautiously in patients with arterial disease. Total contact casting is highly effective for offloading pressure in diabetic foot ulcers, though it limits mobility and requires expertise to apply [59]. When wounds are extensive or fail to heal, surgical interventions like skin grafts or flap surgeries are often necessary to restore skin integrity, despite being more invasive and resource-intensive. While these traditional treatments form the backbone of wound care, they often fall short in addressing underlying issues such as chronic inflammation, infection persistence, and poor blood supply, which are common barriers in chronic wounds. To provide a clearer picture of these conventional approaches, Table 4 outlining the different traditional wound healing methods, along with their benefits and drawbacks, is included.

## 3. Characteristics of an Ideal Wound Healing System

An ideal wound healing system must be versatile enough to handle different types of wounds. Because wounds vary widely—from acute surgical cuts to chronic ulcers—the dressing should adapt to different sizes, characteristics, and levels of fluid. Smart materials like stimuli-responsive hydrogels are designed to change their properties based on the wound environment, making them suitable for many clinical uses [84,85]. It is worthwhile to mention that Stimuli-responsive or “smart” hydrogels are advanced biomaterials that modify their structure or function in response to local wound conditions such as pH, temperature, ROS, glucose, or enzymatic activity [86]. They are derived from natural polymers like chitosan, alginate, gelatin, and hyaluronic acid [87], or synthetic ones such as PNIPAAm, poly(alkylacrylamide), poly(alkylmethacrylate), and PEG [87]. Crosslinked into 3D networks, these hydrogels swell or degrade in response to stimuli, enabling precise drug delivery and environmental adaptability [88]. Compared to traditional dressings, they offer real-time responsiveness, ROS scavenging, and enhanced tissue regeneration, making them ideal for chronic wounds, diabetic ulcers, and burns [89].

Maintaining an optimal moisture equilibrium is critical for wound healing because a moist environment facilitates cell migration, accelerates epithelialization, and avoids the risk of desiccation. Advanced dressings (particularly hydrogels and certain foam formulations) have been reported to keep the environment moist while still allowing for gas transfer, which is essential for the wound’s healing processes. For example, recent advances in hydrogel-based dressings have highlighted their capability to maintain an optimal environment for moisture, thereby promoting faster healing [90]. Effective wound protection is essential. An ideal wound care system should act as a physical barrier, shielding the wound from mechanical injury and external contaminants. This is often achieved using dressings made from natural or synthetic polymers, which not only protect the tissue but also help regulate the wound’s microenvironment [91]. Recent reviews of modern wound care materials consistently highlight the importance of these dressings in preventing secondary infections and actively supporting the healing process. Infection control is a key component of effective wound management. Microbial colonization is a common barrier to healing, especially in complex and chronic wounds. To address this, antimicrobial agents like silver, iodine, and copper are often incorporated into wound dressings. Systematic reviews and meta-analyses have shown that these dressings can significantly reduce microbial load and lower the risk of infection [92]. Efficient oxygen exchange is vital for cellular metabolism and tissue regeneration during wound healing. Higher oxygen levels stimulate new blood vessel growth and enhance the cellular activities necessary for repair. Both hyperbaric oxygen therapy and topical oxygen delivery methods have been shown to increase oxygen diffusion into the wound bed, promoting the formation of granulation tissue [93,94]. Another key aspect of wound care is managing excess exudate. Wounds that produce a lot of fluid need dressings that can absorb moisture quickly while keeping the wound environment balanced. Advanced foam dressings and superabsorbent spacer fabrics have been specially designed for this, offering faster absorption and better fluid control than traditional materials [95]. Similarly, dressings must be non-adherent and easy to remove without causing pain or damaging healing tissue. Biomaterials like carrageenan-based compounds are designed to stick gently enough to protect the wound but release easily during dressing changes, minimizing discomfort and tissue trauma [96]. Biocompatibility and non-toxicity are crucial for an ideal wound healing system. Materials must avoid triggering harmful immune reactions and support the survival of healthy cells. Recent developments in biodegradable synthetic polymers and natural biopolymers offer excellent options that meet these criteria, making them well-suited for wound dressings [97,98]. Ease of application and wound monitoring are important practical factors in wound care. Modern dressings increasingly include wearable sensors that continuously track pH, temperature, and moisture, offering real-time insights into the wound’s condition. This technology not only makes applications easier but also enables remote monitoring, enhancing personalized treatment [99,100]. Finally, the ability to deliver therapeutic agents directly to the wound site is an important feature, as it allows targeted treatment that can enhance healing and reduce infection [101]. Incorporating drug-delivery systems—like hydrogels loaded with antibiotics, anti-inflammatory agents, or growth factors—allows wound dressings to actively support healing by reducing inflammation and promoting tissue regeneration. Recent studies on Aloe vera-based and chitosan hydrogels have shown effective, sustained release of these therapeutic agents, which plays a crucial role in improving wound repair [102].

**Figure 1:**
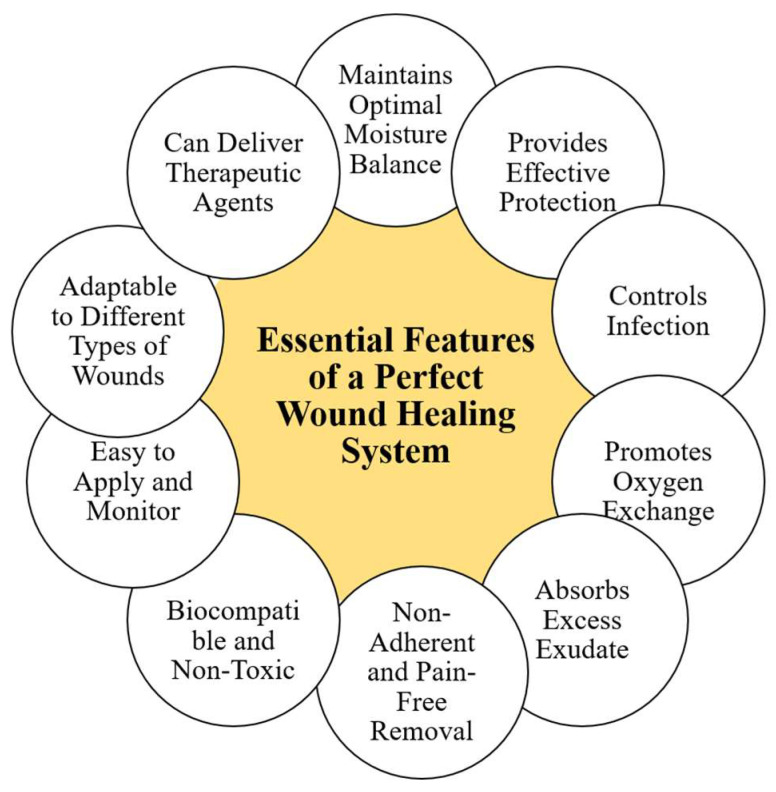
Key Properties for an Optimal Wound Healing System.

In summary, the ideal wound healing system combines multiple key functions: maintaining optimal moisture balance, providing strong protection, controlling infection effectively, enhancing oxygen exchange, absorbing excess exudate, allowing painless and non-adherent removal, ensuring biocompatibility and non-toxicity, offering ease of application and monitoring, adapting to various wound types, and actively delivering therapeutic agents. Together, these features create a comprehensive healing environment that accelerates tissue repair, reduces complications, and improves patient quality of life. Figure 1 summarizes the essential features for an ideal wound healing system.

## 4. Importance of Hydrogels in the Wound Healing System

Standard treatments like dry dressings, non-adherent films, hydrocolloids, and basic antimicrobial ointments often fall short because they cannot address all the factors that contribute to chronic or complex wounds [26]. Because chronic wounds involve multiple challenges—such as infection, oxidative stress, and poor blood flow—new treatments are necessary. This has sparked interest in advanced materials like hydrogels, which can keep the wound moist, deliver drugs or growth factors over time, and respond dynamically to the wound’s microenvironment. Hydrogels are three-dimensional, soft, water-rich polymer networks. Their high water content helps keep wounds moist, which supports cell migration, proliferation, and tissue regeneration. This moist environment not only speeds healing but also typically reduces scarring. Additionally, hydrogels act as a physical barrier, protecting wounds from bacteria and contaminants while allowing balanced exchange of gases, nutrients, and moisture [103,104].

A fundamental property of hydrogels is their swelling behavior in aqueous environments. Upon immersion in water or physiological fluids, hydrogels absorb significant amounts of liquid and undergo volumetric expansion. This phenomenon is primarily driven by the presence of hydrophilic functional groups—such as hydroxyl, carboxyl, and amide moieties—along the polymer backbone, which interact with water molecules through hydrogen bonding. The extent of swelling is governed by multiple parameters, including the chemical composition of the polymer network, the degree of crosslinking, and external environmental factors such as pH, temperature, and ionic strength [105]. For example, hydrogels composed of polyacrylic acid or alginate exhibit pH-responsive swelling behavior. In alkaline environments, the carboxyl groups within the polymer chains undergo ionization, leading to increased electrostatic repulsion and osmotic pressure within the network, thereby enhancing the degree of swelling [106,107].

pH-sensitive hydrogels, derived from both synthetic and natural polymers, demonstrate dynamic swelling behavior in response to variations in the wound microenvironment, particularly fluctuations in pH. This responsiveness is highly advantageous in wound healing, as the local pH can shift from acidic in infected or inflamed wounds to neutral or slightly alkaline in healing tissues [108]. These hydrogels swell, degrade, or release therapeutic agents in response to such pH changes, making them ideal candidates for smart wound dressings [109]. The swelling mechanism is governed by the ionization of functional groups within the polymer chains: at acidic pH, carboxyl groups (–COOH) remain protonated, minimizing electrostatic repulsion and limiting swelling; conversely, at neutral or alkaline pH, these groups deprotonate to –COO^−^, increasing repulsion and water uptake, thereby enhancing swelling [110]. This pH-induced swelling facilitates controlled drug release, improves oxygen diffusion, and maintains a moist wound environment, all of which contribute to accelerated wound healing [111]. Specifically, targeted drug release can be achieved by designing hydrogels that swell and release antimicrobials or growth factors at defined pH levels, enabling precise therapeutic action. In infected wounds, where the pH becomes acidic, swelling is triggered, activating the release of antimicrobial agents and aiding in infection control. Simultaneously, the swelling behavior helps regulate wound moisture, maintaining hydration and promoting epithelialization. Furthermore, the inherent biodegradability of many pH-sensitive hydrogels allows for gradual degradation in response to pH changes, enabling their natural removal without the need for frequent dressing changes. Synthetic hydrogels, such as those based on poly(acrylic acid) (PAA), poly(methacrylic acid), and PLGA, offer high pH sensitivity, tunable mechanical strength, and sustained drug delivery capabilities [112]. In contrast, natural hydrogels—such as chitosan, which swells in acidic conditions and possesses antimicrobial properties; alginate, which responds to both pH and ionic changes; and gelatin, which supports cell adhesion and is both thermo- and pH-responsive—offer biocompatibility and biodegradability [113]. These properties collectively enhance tissue regeneration and healing outcomes [114].

Hydrogels are viscoelastic materials that exhibit a combination of elastic, solid-like and viscous fluid-like behavior, making them particularly attractive for biomedical applications such as wound healing and tissue engineering. The polymer’s chemical structure governs their mechanical performance, the type and degree of crosslinking, and the presence of reinforcing agents or nanofillers that improve mechanical strength and load-bearing capacity [115]. Chemically crosslinked hydrogels, such as those based on polyethylene glycol diacrylate (PEGDA), form covalent bonds within their polymer networks, resulting in superior mechanical integrity and structural stability compared to physically crosslinked hydrogels, which rely on weaker non-covalent interactions such as hydrogen bonding or ionic forces [116]. Notably, double-network (DN) hydrogels, comprising two interpenetrating polymer networks with contrasting mechanical profiles, exhibit exceptional toughness and elasticity due to efficient energy dissipation under mechanical stress—attributes that make them ideal for applications in load-bearing tissues like cartilage and ligaments [117]. Another critical feature is their tunable porosity and permeability; by adjusting polymer concentration, crosslinking density, and incorporating porogens or templating agents, the pore size and distribution can be precisely engineered. This interconnected porous architecture facilitates the diffusion of oxygen, nutrients, and therapeutic agents, while also promoting cell infiltration and tissue integration—crucial for both drug delivery and regenerative medicine [118,119]. Furthermore, the viscoelastic nature of hydrogels enables them to replicate the mechanical environment of native extracellular matrix (ECM), thereby supporting essential cellular activities such as adhesion, migration, proliferation, and angiogenesis [120]. Their ability to dissipate mechanical stress minimizes damage during dressing changes, while their conformability and injectability enhance integration with irregular wound geometries. Ultimately, the selection of chemically versus physically crosslinked hydrogels should be informed by the clinical context: the former offers enhanced strength and durability for chronic or load-bearing wounds, whereas the latter provides superior adaptability and biocompatibility, suitable for acute or sensitive applications.

Hydrogels also exhibit thermal properties that are particularly advantageous for applications such as injectable systems and smart drug delivery platforms. Certain hydrogels are thermoresponsive, undergoing a reversible sol–gel phase transition at specific temperatures. This behavior is typically driven by changes in polymer–solvent interactions, enabling the hydrogel to remain in a liquid state at room temperature and transition to a gel upon exposure to physiological temperatures, thus facilitating minimally invasive administration and in situ gelation [121]. A well-characterized example is poly(N-isopropylacrylamide) (PNIPAM), which exhibits a lower critical solution temperature (LCST) near 32 °C. Below the LCST, PNIPAM hydrogels are highly hydrated and swollen due to favorable interactions between the polymer and water. Above this temperature, the polymer chains become hydrophobic, leading to network collapse and expulsion of water [122,123]. This thermoresponsive behavior is particularly advantageous for the design of injectable hydrogels that undergo in situ gelation upon reaching body temperature, enabling minimally invasive administration and localized delivery of therapeutics. The chemical stability and biodegradability of hydrogels are largely dictated by the polymer backbone composition and the nature of the crosslinking. Synthetic hydrogels, such as those based on polyethylene glycol (PEG), typically exhibit high chemical stability and resistance to enzymatic degradation, rendering them ideal candidates for long-term implantable devices [124]. In contrast, Natural polymers such as chitosan, alginate, collagen, hyaluronic acid, and cellulose are not just passive scaffolds, they actively participate in wound repair through biochemical signaling, immune modulation, and cellular integration [125]. Their synergy arises from the interplay of structural, chemical, and biological functions, which collectively enhance healing outcomes. Natural polymers play a pivotal role in wound healing by creating an optimal environment that supports tissue regeneration, regulates immune responses, and enhances cellular integration. Polymers such as alginate and hyaluronic acid help maintain a moist wound bed, which is essential for cell migration and proliferation. Their gel-like structure mimics the extracellular matrix (ECM), providing a scaffold that facilitates tissue repair [126]. Additionally, these materials are biodegradable and biocompatible, breaking down into non-toxic byproducts and integrating seamlessly with host tissues without provoking adverse immune reactions. Beyond structural support, natural polymers actively modulate immune responses. Many contain bioactive compounds or can be infused with herbal agents like curcumin and quercetin, which suppress pro-inflammatory cytokines and oxidative stress, thereby promoting a smoother transition from inflammation to proliferation. Chitosan, in particular, encourages M2 macrophage polarization—associated with tissue repair—while inhibiting the pro-inflammatory M1 phenotype [127]. Furthermore, these polymers exhibit antioxidant properties, scavenging reactive oxygen species (ROS) that are elevated in chronic wounds and contribute to delayed healing. On a cellular level, polymers such as collagen and gelatin provide integrin-binding sites that enhance cell adhesion and migration, enabling keratinocytes, fibroblasts, and endothelial cells to repopulate the wound bed [128]. Hyaluronic acid also stimulates angiogenesis by promoting endothelial cell proliferation and migration, ensuring adequate vascularization for nutrient delivery and waste removal. Some polymers are engineered to release growth factors or recruit stem cells, accelerating regeneration and improving the structural integrity of the healed tissue. The synergy among these polymers—especially when blended, such as chitosan with alginate or collagen—amplifies their individual benefits, resulting in enhanced mechanical strength, antimicrobial activity, and cellular compatibility [129]. This synergistic effect enables the development of customizable wound dressings tailored to various wound types, including acute, chronic, infected, or ischemic wounds, ultimately improving healing outcomes and patient care. Collectively, these natural polymers contribute not only to maintaining an optimal healing environment but also to modulating immune responses and enhancing cellular integration within the wound bed.

Surface properties and biointeractions are also crucial, especially for biomedical applications. Hydrogels are typically hydrophilic, which reduces protein adsorption and minimizes immune responses. However, their surfaces can be modified with peptides, growth factors, or other bioactive molecules to enhance cell adhesion and proliferation [130,131]. For example, coating polyester-based materials with hydrogel layers has been shown to improve protein release and cell viability. Researchers have further enhanced hydrogels by developing bioactive or composite formulations. For example, certain hydrogels are functionalized with antimicrobial agents—such as silver nanoparticles, honey, essential oils, or antibiotics—to provide effective infection control [132]. Other hydrogels are engineered to deliver growth factors that promote angiogenesis and tissue regeneration. Additionally, some hydrogels serve as cell carriers, encapsulating viable cells—such as stem cells or fibroblasts—to enhance tissue repair and accelerate the healing process [133]. In addition, nanocomposite hydrogels incorporate nanoparticles—such as zinc oxide or graphene oxide—to simultaneously enhance mechanical strength and antibacterial efficacy, making them highly effective as wound dressings.

### Hydrogels for Treatment of Burn Wounds

Burn wounds present significant clinical challenges due to their high risk of infection, intense pain, and propensity for scarring. Hydrogels—both synthetic and natural—have emerged as advanced platforms for burn wound management due to their ability to maintain a moist healing environment, provide a cooling effect, deliver therapeutic agents, and mimic the extracellular matrix (ECM) [134]. Unlike conventional gauze dressings, which can dry out wounds, adhere to tissue, and cause pain upon removal, hydrogels form non-adherent, hydrophilic, three-dimensional polymeric networks that conform gently to the wound bed without disrupting newly formed tissue. This minimizes trauma during dressing changes and is especially beneficial in pediatric or extensive burn cases.

Hydrogels are particularly effective in treating second-degree burns, where moisture balance, infection control, and tissue regeneration are essential. Their high-water content facilitates gradual evaporation upon application, resulting in an immediate thermoregulatory cooling effect that alleviates pain, reduces local temperature, and limits further thermal injury [135,136]. This effect is most beneficial in the initial hours post-injury and helps preserve surrounding viable tissue, thereby enhancing natural regeneration [137,138]. Moreover, maintaining a moist environment significantly accelerates re-epithelialization by promoting keratinocyte migration, cellular proliferation, and matrix remodeling [139,140].

In addition to physical wound protection, hydrogels serve as versatile drug delivery vehicles. Their porous structure allows for the incorporation and localized, sustained release of bioactive agents, including antibiotics, anti-inflammatory compounds, and growth factors. This sustained release prolongs therapeutic efficacy at the wound site, reducing infection risk, pain, and the need for systemic treatments. Incorporation of antimicrobial agents such as silver nanoparticles or zinc ions has shown to enhance infection control, while natural bioactive substances like honey and plant extracts further support tissue regeneration and immunomodulation [141]. The localized drug delivery provided by hydrogels not only enhances therapeutic effectiveness but also minimizes systemic side effects.

Synthetic hydrogels—based on materials such as polyvinyl alcohol (PVA), polyethylene glycol (PEG), and polyacrylamide (PAAm)—offer tunable mechanical properties, elasticity, and controlled drug release, though they may require chemical crosslinkers that pose cytotoxicity risks and limit biodegradability [142]. In contrast, natural hydrogels—derived from polymers such as chitosan, alginate, gelatin, and hyaluronic acid—are inherently biocompatible and biodegradable, with intrinsic antimicrobial and anti-inflammatory properties. These materials also support ECM mimicry and cellular adhesion, although they often possess lower mechanical strength and variable degradation kinetics. Notably, natural hydrogels like chitosan and alginate have demonstrated superior efficacy in burn care by accelerating healing and reducing infection rates. For example, Huang et al. developed a sulfated alginate hydrogel loaded with Prussian blue nanozymes that modulates macrophage polarization and reduces excess ROS in wounds. Mimicking sulfated glycosaminoglycans in the ECM, the alginate binds inflammatory chemokines like IL-8 and MCP-1, limiting immune cell infiltration. It also promotes the M1-to-M2 macrophage transition. In mice with deep second-degree burns, this targeted immunomodulation enhanced neovascularization, granulation tissue, collagen deposition, and wound healing [143]. Calcium alginate hydrogels have been shown to accelerate the healing of second-degree burns. In another study, an alginate hydrogel containing cerium oxide nanoparticles matrix was developed and In vitro studies demonstrated that the wound dressing based on this system exhibited favorable antioxidant properties and displayed hemocompatibility and biocompatibility. Animal studies conducted on a rat full-thickness skin wound model showed that the alginate hydrogel-based wound dressing effectively accelerated the wound healing process [144]. Chitosan’s intrinsic antimicrobial properties make it a promising candidate for treating infected burn wounds. Aldakheel et al. utilized green tea leaf extract as a natural reducing agent to synthesize silver nanoparticles (AgNPs), which were subsequently incorporated into chitosan (Ch)-grafted polyvinyl alcohol (PVA) hydrogels via microwave irradiation. The resulting AgNPs/Ch-PVA hydrogel was evaluated for wound-healing efficacy through both in vitro (fibroblast cell culture) and in vivo (rat model) studies. The hydrogel demonstrated notable antibacterial activity against *Escherichia coli* and *Staphylococcus aureus*, as confirmed by the agar diffusion method, indicating its potential for effective management of infected wounds [145]. Gelatin-methacrylate (GelMA) hydrogels have been shown to promote revascularization and epithelial regeneration. The GelMA hydrogels were applied to rat burn wounds and resulted in significant collagen alignment and reduced hypertrophic scarring. Overall, hydrogel-based burn dressings provide a multifunctional platform that combines mechanical protection, pain relief, infection control, and enhanced tissue regeneration. Their customizable properties and drug-loading capabilities make them particularly valuable in treating complex or chronic burn wounds, significantly improving patient outcomes compared to traditional dressings.

Hydrogels have demonstrated remarkable efficacy in laboratory settings, particularly in promoting wound healing through moisture retention, drug delivery, and biocompatibility. However, translating these findings into clinical practice requires more robust human data. Recent meta-analyses and clinical trials involving over 1700 patients have shown that hydrogel dressings significantly improve wound closure rates and reduce healing time compared to conventional treatments [146]. Additionally, over 100 hydrogel-based products—both injectable and topical—have received regulatory approval, with more than 210 active clinical trials currently underway [147]. These studies underscore the real-world potential of hydrogels but also highlight the need for comparative effectiveness research and long-term outcome data. Table 5 shows some commercial and clinical hydrogels that are used for wound treatment.

These commercial products are now standard-of-care in many settings, attesting to the translation of hydrogel science to widespread medical practice.

## 5. Hydrogel Composition and Types

Hydrogels are generally classified based on their origin—either natural or synthetic—and the mechanism by which their polymer networks are formed, which can be either physical or chemical crosslinking. In many instances, hybrid hydrogel systems are engineered to integrate the advantageous properties of both natural and synthetic components. These systems often employ multiple crosslinking strategies to enhance the mechanical, structural, and functional performance of the hydrogel [155].

### 5.1. Classification Based on Compositions

#### 5.1.1. Natural Hydrogels

Natural hydrogels, derived from biologically sourced polymers such as chitosan, alginate, and collagen, are widely valued in biomedical fields—including tissue engineering, wound healing, and drug delivery—due to their excellent biocompatibility, biodegradability, and ability to mimic the extracellular matrix (ECM) [156]. Their natural origin imparts unique bioactive features, such as cell adhesion motifs and enzymatically degradable regions, which facilitate cell attachment, proliferation, and tissue remodeling. However, despite these advantages, their clinical translation and long-term application remain limited by several critical challenges. The most notable of these is poor mechanical strength, which restricts their use in load-bearing or dynamic environments like cartilage or muscle tissue. Other limitations include batch-to-batch variability, limited shelf stability, uncontrolled degradation rates, poor adhesion to moist or irregular tissue surfaces, restricted functionalization potential, and, in some cases, immunogenic responses, especially when animal-derived components are used. Fortunately, various strategies have been developed to address these issues [157]. Chemical crosslinking, using agents like genipin or carbodiimide, introduces covalent bonds that enhance network density and stiffness [158]. Physical crosslinking methods, such as ionic or hydrogen bonding, offer reinforcement without toxic additives, making them suitable for in vivo use [159]. Additionally, nanocomposite hydrogels, formed by incorporating nanoparticles like nanocellulose, silica, or graphene oxide, significantly improve mechanical resilience while maintaining biological compatibility. Tuning polymer concentration and molecular weight further allows customization of elasticity and stiffness to match specific tissue requirements [160].

#### 5.1.2. Synthetic Hydrogels

The precisely controlled chemical composition and network structure of synthetic hydrogels offer significant advantages such as reproducibility, mechanical strength, and adjustable physical properties. This control enables customization of factors like swelling, degradation rate, and stiffness, making synthetic hydrogels particularly useful for applications requiring high structural integrity and consistent performance [161]. Although synthetic hydrogels generally lack natural bioactive signals, they are often modified with peptides, growth factors, or other biologically active molecules to enhance cellular compatibility and biological function [162]. These modifications improve the ability of synthetic hydrogels to support cell adhesion, growth, and tissue integration. Synthetic hydrogel-based smart materials have demonstrated significant potential in enhancing oxygen diffusion in wound dressings, a crucial factor for accelerating tissue regeneration, particularly in hypoxic or chronic wound environments where oxygen-dependent processes such as cellular metabolism, angiogenesis, and collagen synthesis are compromised [163]. These hydrogels can be engineered with functional components that facilitate oxygen transport or controlled oxygen release. Key strategies include embedding oxygen nanobubbles (ONBs), which provide a sustained release of oxygen directly into the wound bed; designing porous hydrogel networks that are permeable to ambient oxygen; and incorporating oxygen-generating compounds such as calcium peroxide or magnesium peroxide that react with wound exudate to release oxygen [164]. These approaches help maintain oxygenation in hypoxic tissues, promoting cellular proliferation and vascularization. An illustrative example is the development of a Carbopol-based hydrogel system incorporating ONBs and collagenase, which released 12.08 ± 0.75 μg/mL of oxygen and maintained delivery for up to three weeks while preserving enzymatic collagenase activity for 17 days [165]. This system supported autolytic debridement, enhanced cell migration in vitro, and significantly improved re-epithelialization, angiogenesis, and follicular regeneration in vivo, highlighting the promise of oxygenating smart hydrogels in advanced wound care.

However, despite these benefits, synthetic hydrogels also have notable drawbacks. Their absence of natural bioactivity can limit cell recognition and integration unless heavily modified, which increases complexity and cost. Additionally, the breakdown products of some synthetic polymers may cause cytotoxicity or trigger immune responses. The synthetic nature of these materials may also hinder their ability to fully replicate the dynamic and complex environment of native extracellular matrices [161]. Moreover, methods for adding bioactive molecules may not always distribute them evenly, leading to inconsistent biological responses.

#### 5.1.3. Hybrid Hydrogels

To overcome the limitations of natural and synthetic hydrogels, hybrid hydrogels have emerged as next-generation biomaterials. These are engineered by combining natural and synthetic polymers or integrating both physical and chemical crosslinking mechanisms to synergistically enhance performance. Such hybrid systems exhibit improved mechanical strength, enhanced bioactivity, tunable degradation rates, and better mimicry of the native ECM [166,167]. Incorporation of functional additives, such as nanoparticles (e.g., silver, zinc oxide) or vesicles (e.g., exosomes), further imparts antimicrobial properties and promotes tissue regeneration, expanding the applicability of hybrid hydrogels across biomedical domains. These materials are particularly valuable in drug delivery, where they offer controlled, localized release through stimuli-responsive mechanisms (e.g., pH, temperature, light, or mechanical force), and in regenerative medicine, where their adjustable properties support both soft and hard tissue repair. As highlighted in recent perspectives, hybrid hydrogels effectively bridge the gap between synthetic precision and biological functionality, representing a versatile platform for biosensors, actuators, wearable devices, personalized medicine, and smart therapeutic systems [168].

### 5.2. Classification Based on Network

#### 5.2.1. Physical Hydrogels

Hydrogels can also be classified according to the nature of the interactions that stabilize their polymeric networks. Physical hydrogels are formed through non-covalent interactions such as hydrogen bonding, hydrophobic interactions, ionic interactions, and van der Waals forces. These reversible and dynamic interactions confer stimulus-responsive behavior and self-healing properties to physical hydrogels. However, due to the transient nature of these bonds, physical hydrogels generally exhibit lower mechanical strength and stability compared to chemically crosslinked hydrogels. Owing to the reversible nature of their non-covalent bonds, physical hydrogels frequently exhibit stimuli-responsive behavior and self-healing properties. These dynamic interactions allow the polymer network to reorganize and adapt in response to environmental stimuli such as pH, temperature, or ionic strength. This responsiveness makes physical hydrogels especially suitable for applications that demand adaptability, controlled release, or reversible structural changes [169]. The reversible nature of these interactions enables the hydrogel’s structure to dynamically change in response to external stimuli such as temperature, pH, or ionic strength [170,171]. This capacity is especially advantageous in drug delivery applications, where controlled release is critical. The ability of physical hydrogels to form gels under mild conditions makes them particularly suitable for encapsulating sensitive biomolecules and living cells, preserving their functionality and viability during the encapsulation process [172].

#### 5.2.2. Chemical Hydrogels

Chemical hydrogels are formed through covalent crosslinking, resulting in permanent bonds between polymer chains under physiological conditions. This covalent network imparts greater mechanical strength and stability, producing hydrogels that are typically more robust and durable compared to those formed by physical interactions [173]. Chemical crosslinking is commonly achieved through the use of initiators, crosslinking agents, or photopolymerization techniques. Hydrogels formed via these covalent bonds exhibit enhanced structural stability and resistance to dissolution, making them particularly suitable for applications that require a persistent and stable scaffold, such as tissue engineering and long-term biomedical implants [174,175]. However, because the crosslinking reactions are typically irreversible, chemical hydrogels tend to be less responsive to environmental stimuli compared to their physically crosslinked counterparts [176].

## 6. Importance of Natural Hydrogels in Wound Healing

In wound healing applications, natural, synthetic, and hybrid hydrogels each provide distinct benefits, but natural hydrogels show several key advantages, especially in biological performance. Natural hydrogels, derived from biopolymers like chitosan, alginate, and gelatin, closely imitate the extracellular matrix (ECM), supporting cell adhesion, migration, and proliferation—essential steps for effective tissue regeneration [177]. Their excellent biocompatibility, biodegradability, and ability to keep a moist wound environment make them perfect for speeding up healing and reducing inflammation. Additionally, some natural hydrogels have inherent antimicrobial and hemostatic properties, which are especially useful for infected or chronic wounds. In Table 6, we give a brief overview of different natural hydrogels.

### 6.1. Polysaccharide-Based Hydrogels

Polysaccharide-based hydrogels are notable for their exceptional ability to retain large amounts of water. Their customizable structural networks make them highly effective in fields like controlled substance delivery, wound care, and sensor technologies. These hydrogels maintain a moist environment conducive to healing while also providing a protective barrier that prevents bacterial invasion. Furthermore, they possess suitable mechanical strength, are biodegradable, and enable the regulated release of medications, all of which contribute to their successful application in therapeutic settings.

#### 6.1.1. Alginate-Based Hydrogels

Alginate, a linear polysaccharide derived from seaweed, consists of homopolymeric sequences of α-L-guluronate (G) and β-D-mannuronate (M) units. Due to its biocompatibility and versatile modifiability, alginate is extensively used to create hydrogels for wound healing applications [190]. A key feature of alginate that enables hydrogel formation is the ability of its G-blocks to crosslink with divalent cations such as Ca^2+^, Mg^2+^, and Ba^2+^ [191] Consequently, factors like the ratio of M to G units, length and sequence of G-blocks, and overall molecular weight significantly influence the physical properties of alginate-based hydrogels. Chang et al. developed a composite hydrogel combining bioglass (BG) and oxidized sodium alginate (OSA), using adipic acid dihydrazide (ADH)-modified γ-polyglutamic acid (γ-PGA) as the cross-linker. This hydrogel supports wound closure and healing through three mechanisms: (a) alkaline ions released from BG promote imine bond formation between the hydrogel and tissue, (b) Ca^2+^ ions from BG enhance adhesion between the hydrogel and implantable biomaterials by chelating with carboxyl groups in alginate, and (c) silicon ions released accelerate blood vessel formation in the wound area [192]. Sui et al. developed a nanoporous, mechanically robust, and compositionally gradient hydrogel film by diffusing low molecular weight chitosan into a solution of high molecular weight sodium alginate. This engineered film exhibits rapid and programmable shape transformation in response to external stimuli such as variations in temperature, ionic strength, and pH conditions [193]. In a related advancement, Hytönen and collaborators designed a nanocellulose-reinforced alginate-based hydrogel suitable for 3D printing applications. The addition of nanocellulose significantly enhanced the rheological properties of the printing paste, effectively preventing structural collapse during printing and preserving the fidelity of printed constructs, thereby demonstrating potential utility in wound healing frameworks [194]. Furthermore, introducing various ions into alginate hydrogels can impart additional functionalities. Studies have explored doping gels with ions such as Rb^+^, Cu^2+^, Zn^2+^, and Sr^2+^ to enhance their antibacterial properties.

In addition, alginate material shows high aqueous solubility, rapid gelation kinetics, and excellent plasticity. It is extensively utilized in the formulation of injectable hydrogels for biomedical applications. For example, Zhao et al. [195] reported a novel double-network hydrogel composed of xanthan gum (XG) and dopamine-modified oxidized sodium alginate (OSA-DA), intended for applications in wound dressing and skin-interfaced biosensing. In this hydrogel, XG forms the foundational polymer matrix through hydrogen bonding interactions, while dopamine moieties covalently bond to OSA via Schiff base linkages, imparting strong tissue adhesion. The incorporation of 45S5 bioactive glass facilitates the controlled release of Ca^2+^ ions, which interact with alginate chains to sustain the hydrogel’s mechanical integrity over time, enabling prolonged release of marine polysaccharides. The hydrogel’s self-healing and injectable nature allows it to conform to wounds of irregular geometry or adhere to skin for sensor-based monitoring. Furthermore, its ionic conductivity enables real-time biosensing functions, establishing a platform for multifunctional hydrogel dressings that integrate therapeutic and diagnostic capabilities.

In the context of oxidative stress around wound sites, elevated levels of reactive oxygen species (ROS) are known to impair tissue regeneration by damaging cellular components such as DNA and proteins [196]. Cannabinol (CBD) has demonstrated the ability to inhibit superoxide production by targeting NOX1, NOX4, and xanthine oxidase (XO), thereby reducing ROS generation [197]. Zinc ions (Zn^2+^) contribute additional benefits, offering broad-spectrum antimicrobial activity and promoting angiogenesis by upregulating vascular endothelial growth factor (VEGF) gene expression [198]. Building on this understanding, Zheng et al. [199] synthesized a multifunctional hydrogel by incorporating CBD and Zn^2+^ into a sodium alginate matrix, resulting in a CBD-loaded zinc alginate (CBD/Alg-Zn) hydrogel. In vitro characterization revealed excellent anti-inflammatory, antioxidant, and antibacterial properties, positioning CBD/Alg-Zn as a promising candidate for advanced wound dressing applications.

The integration of bioactive mineral components into polymeric scaffolds remains a significant challenge in regenerative medicine due to concerns regarding cytotoxicity and biomaterial compatibility [200]. Addressing this, Fadeeva et al. [201] developed a novel hydrogel membrane composed of polyvinylpyrrolidone (PVP), sodium alginate (SA), and hydroxyapatite (HA), termed PVP-HA-SA. HA was incorporated via in situ addition into the PVP matrix to improve biological performance. Experimental assessments using dental pulp stem cells (DPSCs) demonstrated that increasing the PVP content enhanced the adhesion properties of the hydrogel while mitigating cytotoxic effects associated with HA incorporation. The resultant PVP-HA-SA membranes exhibited strong cell adhesion, low toxicity, and a marked capacity to promote tissue regeneration, making them suitable candidates for use as wound dressings in clinical settings.

#### 6.1.2. Chitosan-Based Hydrogels

Chitosan, a natural polysaccharide primarily sourced from crustacean exoskeletons, is obtained through the deacetylation of chitin, which is a linear polymer of N-acetylglucosamine. It stands out as the only naturally occurring basic polysaccharide, carrying a positive charge due to the abundance of amino groups along its chains. Thanks to these unique characteristics, chitosan-based materials can retain their form without external influences, relying solely on natural forces such as surface tension and gravity. For instance, a self-healing hydrogel composed of chitosan (CP) and telechelic difunctional poly(ethylene glycol) (DF-PEG) was synthesized. When applied to rat liver injuries, a thrombin-loaded version of this hydrogel (CPT) produced a smooth liver capsule with healthy coloration, demonstrating its promise as an in vivo drug delivery vehicle for wound healing [202]. In another study, Yao et al. employed thermo-responsive chitosan hydrogels to deliver recombinant human stromal cell-derived factor-1 alpha (SDF-1α) for eye injuries. This treatment facilitated faster restoration of the corneal epithelium, improving both its natural structure and function, while simultaneously increasing the local production of critical growth factors necessary for healing [203].

Chitosan’s notable properties, including its antimicrobial effects and low antigenicity, have made it a popular subject for research in drug delivery systems, antibacterial applications, and tissue engineering [204]. Unlike conventional dressings that rely on the release of antibacterial compounds, chitosan-based materials inherently inhibit microbial growth, potentially offering longer-lasting protection with less toxicity to surrounding tissues. Ma and colleagues introduced an injectable, antibacterial conductive hydrogel made from glycidyl methacrylate-functionalized quaternized chitosan (QCSG) combined with carbon nanotubes (CNTs) which was designed for controlling severe noncompressible bleeding and promoting wound healing. This hydrogel exhibited strong mechanical properties, fast shape recovery triggered by contact with blood, rapid absorption rates, and high blood uptake capacity [205].

A critical consideration in the development of chitosan (CS)-based hydrogels for use as biomedical dressings is their biocompatibility and acceptance by the host tissue. To enhance these properties, oxidized polysaccharides—such as oxidized glucans—have been employed as environmentally friendly bio-crosslinking agents. Of particular note is the application of the Schiff base reaction, which involves the formation of reversible imine bonds, for the fabrication of injectable, self-healing hydrogels [206]. Chen et al. [207] reported the synthesis of a hydrogel dressing composed of CS and oxidized konjac glucomannan (OKGM), employing the Schiff base reaction as the crosslinking mechanism. In this system, glucan undergoes oxidation to produce OKGM, which subsequently reacts with amino groups on chitosan to form a hydrogel via dynamic imine bonding. The resulting hydrogel exhibits notable self-healing behavior and injectability, attributed to the reversible nature of the Schiff base bonds. Despite the advantages offered by this crosslinking approach, such as mild reaction conditions and reversible bonding, limitations remain. The rapid in situ gelation, dynamic bond reversibility, and sensitivity to environmental pH can compromise the hydrogel’s mechanical robustness and tissue adhesion, thereby constraining its clinical applicability [208]. To address these challenges, sprayable hydrogel films have been developed, providing effective protection against infection for large and irregularly shaped wounds. For instance, Wu et al. [209] introduced a method wherein chondroitin sulfate (CSA) was oxidized to yield aldehyde-functionalized CSA (OHC-CSA). Dopamine (DA), known for its strong adhesive properties, was subsequently grafted onto OHC-CSA to form OHC-CSA-DA. This modification improved both the mechanical strength and tissue adhesion of the hydrogel matrix. To further enhance the functional properties of the hydrogel, horseradish peroxidase (HRP) and hydrogen peroxide (H_2_O_2_) were employed to catalyze polyphenol-mediated crosslinking, which was integrated into the Schiff base system to fine-tune the gelation kinetics [210]. This dual crosslinking approach significantly enhanced the biological performance of the hydrogel, including improved antibacterial activity and hemostatic efficacy. Ultimately, a novel multifunctional double-network hydrogel dressing was developed, integrating enzyme-mediated crosslinking, Schiff base chemistry, and hydrogen bonding interactions. The resultant hydrogel exhibited a suite of desirable characteristics, including self-healing capability, high adhesiveness, and sprayability. Both in vitro and in vivo evaluations confirmed the hydrogel’s superior biocompatibility and hemostatic performance, as well as its capacity to accelerate wound healing. These findings suggest a promising avenue for the development of next-generation chitosan-based hydrogel dressings with enhanced functional properties.

#### 6.1.3. Hyaluronic Acid-Based Hydrogels

Hyaluronic acid consists of repeating disaccharide units of N-acetyl-D-glucosamine and D-glucuronic acid connected through β-1,4- and β-1,3-glycosidic bonds. It is a major component of connective tissues, including the extracellular matrix and synovial fluid, where it plays critical roles such as water retention, maintaining the extracellular space, regulating osmotic pressure, and providing lubrication [211]. The abundance of carboxyl and hydroxyl groups within hyaluronic acid enables it to readily form both intra- and intermolecular hydrogen bonds when dissolved in water. Hydrogels based on hyaluronic acid are widely regarded as excellent materials for wound healing, due to their biodegradability, biocompatibility, non-toxicity, lack of immunogenicity, and anti-inflammatory effects. For example, Cho et al. developed a hyaluronic acid-based hydrogel patch designed as a ready-to-use tissue adhesive suitable for a broad range of biomedical applications [212]. Li and colleagues reported a method in which hydrazine groups were attached to hyaluronic acid by reacting carboxyl and amino groups. Subsequently, the hydrazine-modified hyaluronic acid was crosslinked with benzaldehyde-functionalized F127 to form micelles containing dynamic acylhydrazone bonds, resulting in a hydrogel formed through micellization and reversible acylhydrazone linkages [213]. Ossipov et al. designed a moldable hydrogel based on hyaluronic acid functionalized with bisphosphonate (BP) groups. Upon addition of silver ions (Ag^+^), immediate gelation occurred via metal–bisphosphonate coordination, forming a hydrogel with potent antimicrobial activity effective against both Gram-positive and Gram-negative bacteria, which is valuable for preventing wound infections [214]. Hyaluronic acid hydrogels can also be engineered to deliver drugs, growth factors, or cells. For instance, a pH-responsive hydrogen sulfide (H_2_S) donor named JK1, synthesized from phenylphosphonothioic dichloride [215], was encapsulated in hyaluronic acid hydrogels. This composite significantly promoted wound healing by encouraging in situ polarization of M2 macrophages. In vivo experiments on skin wounds showed enhanced re-epithelialization, collagen formation, angiogenesis, and cell proliferation with the hyaluronic acid/JK1 hydrogel [216].

An injectable hydrogel system was also developed using hyperbranched multi-acrylated polyethylene glycol (HP-PEG) macromers combined with thiolated hyaluronic acid (HA-SH), serving as a platform for stem cell delivery and retention [217]. Furthermore, Thornton et al. reported a redox-responsive hyaluronic acid hydrogel using aminoethyl disulfide (AED) as a glutathione (GSH)-sensitive crosslinker. This hydrogel provides a cost-effective method for visually detecting target analytes that reflect the redox state of cells and tissues, which can be useful for wound monitoring [218].

Research has shown that natural deep eutectic solvents (DES) exhibit excellent biocompatibility and are highly effective in promoting wound healing, indicating their strong potential in the development of innovative hydrogel wound dressings [219]. In a pioneering study, Li et al. [220] created a new antibacterial hydrogel dressing—DES-DASH@Ag—by incorporating DES (a combination of glucose and choline chloride functioning as hydrogen bond donor and acceptor, respectively) into a lyophilized DASH polymer network. Both glucose and choline chloride not only aid in regenerating skin tissue and preventing infections but also contribute to the formation of silver nanoparticles (AgNPs). These AgNPs, generated in situ through the reduction in dopamine (DA) and stabilized with sodium hyaluronate (SH), demonstrate significant antibacterial properties. As a result, the DES-DASH@Ag hydrogel dressing exhibited outstanding antibacterial activity and biocompatibility, maintaining more than 80% cellular viability. The sponge-like architecture of the DES enhances the hydrogel’s ability to absorb and retain moisture, thereby preserving a moist wound environment and facilitating healing [221]. This positions the material as a promising option for clinical wound care applications.

Photocrosslinking technology has garnered widespread interest for in situ hydrogel formation in wound dressings, mainly due to its rapid reaction rate, minimal invasiveness, and controllable timing [222]. Nonetheless, traditional photoinitiators often suffer from drawbacks like poor solubility in water and cytotoxic effects. Interestingly, it has been discovered that UV-induced crosslinking of hydrogels can proceed without the need for photoinitiators, avoiding the generation of harmful byproducts. Additionally, dual-network hydrogels can simultaneously offer high mechanical strength and toughness, endowing them with multiple advanced properties [223]. Mao et al. [224] introduced a multifunctional dual-network hydrogel (CMC-AZ/HA-NB), made from azide-modified carboxymethyl cellulose (CMC-AZ) and o-nitrobenzyl-modified hyaluronic acid (HA-NB). When exposed to UV light, an aldehyde reaction occurs, producing aldehyde groups that quickly form a hydrogel in situ by reacting with amino groups in CMC. These aldehyde groups also create imine bonds with amino groups in skin tissue, allowing the hydrogel to adhere firmly to the wound surface. Additionally, the azide groups help form a secondary network, improving the hydrogel’s mechanical properties that initially relied on Schiff base mono-crosslinking. To support therapeutic function, amoxicillin was encapsulated as a model drug within the hydrogel, effectively aiding wound healing.

#### 6.1.4. Cellulose-Based Hydrogels

Cellulose, the most abundant renewable polymer on Earth, consists of D-glucopyranose units linked by β-1,4-glycosidic bonds. Its derivatives, valued for biocompatibility, biodegradability, low toxicity, and cost-effectiveness, are widely used in medical research and clinical applications [225]. Mansur et al. developed eco-friendly hydrogels from carboxymethylated cellulose derivatives, which were chemically crosslinked using citric acid and further modified with polyethylene glycol through a sustainable aqueous process [226]. In another study, Tan and colleagues fabricated a chitosan–cellulose composite hydrogel sensitive to pH changes, intended for drug delivery and wound care. This was achieved by first converting hydroxyl groups on carboxymethyl cellulose into aldehydes, which then reacted with amino groups on carboxymethyl chitosan via a Schiff-base mechanism to form the gel network. The antimicrobial agent silver sulfadiazine was incorporated into this matrix, demonstrating controlled release profiles at both acidic (pH 5.5) and alkaline (pH 9.5) environments [227]. A quaternized hydroxyethyl cellulose combined with mesocellular silica foam (MCF) was synthesized into a hydrogel sponge (QHM) via one-step radical graft copolymerization. This sponge rapidly swells upon hydration, concentrating blood components to aid in clotting. With an MCF content of 9.8%, QHM also activates coagulation factors, facilitating hemostasis and improving wound repair outcomes [228]. In another study to develop inherent antibacterial hydrogels, cellulose was isolated from four distinct plant species native to Ecuador and subsequently employed as a matrix for the development of cellulose-based antibacterial hydrogels. The hydrogels synthesized from these natural sources were systematically compared to a reference hydrogel formulated using commercially available cellulose [229]. Comprehensive characterization was performed on both the extracted cellulose powders and the hydrogels obtained from them. Notably, the cellulose extracted from the pear mesocarp (designated as F1) failed to form a stable hydrogel network and was therefore excluded from further experimentation. The study proceeded with hydrogels synthesized from the pear epicarp (F4), tomato (F12), pitahaya (F53), and commercial carboxymethyl cellulose (CMC). These selected hydrogels were evaluated for in vitro antimicrobial efficacy and assessed for potential biomedical application as wound dressings using a pigskin model as a conceptual validation platform. Among the tested samples, the hydrogel derived from pitahaya cellulose (F53) demonstrated superior antibacterial performance, indicating its potential utility in promoting wound healing. The experimental findings imply that the observed antimicrobial effectiveness is likely attributed to intrinsic antifouling properties of the hydrogel matrices, which impede bacterial adhesion and proliferation.

Cellulose-based hydrogels also serve as effective carriers for cell delivery. For instance, bacterial cellulose blended with acrylic acid (BC/AA) was used to create a hydrogel scaffold seeded with human epidermal keratinocytes and dermal fibroblasts, promoting healing in burn injuries [230].

Nanocellulose plays a vital role in enhancing hydrogel properties. Hydrolyzing cellulose fibers yields cellulose nanocrystals (CNs), which are defect-free, rod-shaped crystals exhibiting exceptional mechanical strength (elastic modulus of 130–150 GPa), high specific surface area (up to hundreds of square meters per gram), low density (1.6 g/cm^3^), chemically reactive surfaces, and unique morphology [231]. These features make CNs excellent candidates for both structural reinforcement and crosslinking within hydrogel networks. Chen et al. designed a hydrogel reinforced with these nanocrystals by forming dynamic Schiff base bonds between amine groups on water-soluble carboxymethyl chitosan (CMC) and aldehyde groups on dialdehyde-modified cellulose nanocrystals (DACNC). The DACNCs functioned as dynamic crosslinkers capable of reversible bonding, enabling the hydrogel to self-heal rapidly. Moreover, they acted as reinforcing fillers, significantly increasing the material’s mechanical strength [232].

#### 6.1.5. Dextran-Based Hydrogels

Dextran is mainly composed of D-glucopyranose units connected through α-1,6 glycosidic bonds, with small proportions of α-1,2-, α-1,3-, or α-1,4-linked branches [233]. Similar to other polysaccharides, dextran is highly biocompatible and contains numerous hydroxyl groups that are readily modifiable, making it a valuable material for biomedical use [234,235]. Hydrogels derived from dextran have demonstrated significant enhancement of neovascularization and skin tissue regeneration in animal models [236].

To optimize tissue ingrowth, vascularization, and controlled degradation, Gerecht and colleagues engineered a dextran-based hydrogel by adjusting the proportion of dextran–allyl isocyanate–ethylamine (Dex-AE) and polyethylene glycol diacrylate (PEGDA). Their 3-week in vivo comparison revealed that the hydrogel formulation with an 80:20 ratio of Dex-AE to PEGDA significantly supported dermal regeneration, including full restoration of skin appendages [237]. A novel approach was applied to incorporate both resveratrol (Res) and a vascular endothelial growth factor (VEGF) plasmid into a chemically modified composite hydrogel consisting of dextran, hyaluronic acid (HA), and β-cyclodextrin (β-CD). Resveratrol, known for its anti-inflammatory properties, was encapsulated within the hydrophobic cavity of β-CD, while the VEGF-encoding plasmid DNA (pDNA-VEGF), condensed with polyethyleneimine (PEI), was incorporated into the hydrogel matrix. This complex hydrogel effectively accelerated burn wound healing by reducing inflammation and enhancing microvascular development [238]. Various antibacterial dextran-based hydrogels have also been developed by loading them with silver nanoparticles [239] or the natural compound sanguinarine [240]. Additionally, dextran combined with polyamines has been used to prepare a range of hydrogels exhibiting antibacterial properties [241].

#### 6.1.6. Starch-Based Hydrogels

Starch ranks as the second most plentiful natural biomass and is composed of two glucose-based polymers: amylose and amylopectin. Amylose contains only α-1,4-glycosidic linkages, whereas amylopectin features both α-1,4- and α-1,6-glycosidic bonds [242]. While starch—particularly its modified forms—has found extensive use as a biomaterial [243], it is less favored for hydrogel production compared to other polysaccharides due to its lack of distinctive functional traits beyond biocompatibility. Clinically, starch-based powders have been applied effectively for hemostasis. When creating hydrogels, starch is commonly combined with other polymers to improve performance. Polyvinyl alcohol (PVA) is often chosen as a copolymer due to its hydrophilicity, biocompatibility, and ease of chemical modification [244]. Incorporating active agents into starch-based hydrogels further enhances their utility; antibacterial substances such as copper nanoparticles [245], silver nanoparticles, zeolite nanoparticles [246], and turmeric have been successfully embedded to confer antimicrobial effects to wound dressings. For example, a multifunctional green starch-based hydrogel (Starch@Ca/CGC hydrogel) was developed as a promising wound dressing material, by Xu and co-workers [247]. This hydrogel was fabricated using a multi-cross-linking strategy involving coordination interactions, electrostatic interactions, and both intra- and intermolecular hydrogen bonding. It exhibits excellent mechanical strength, injectability, autonomous self-healing, and strong adhesion, making it suitable for irregular wound surfaces without causing secondary injury. To enhance its therapeutic efficacy, bioactive molecules gallic acid (GA) and carvacrol (CA) were integrated into copper-based nanospheres (CGC NPs) via an ultrasonic-triggered self-assembly and ionic cross-linking process. These nanospheres endowed the hydrogel with controlled near-infrared (NIR) responsive drug release, photothermal antibacterial activity, and antioxidant properties. The hydrogel demonstrated superior antibacterial effects against both Gram-positive and Gram-negative bacteria, including *Staphylococcus aureus*, due to the synergistic effects of the CGC NPs. Biocompatibility and safety were validated through cytotoxicity and hemolysis assays, while in vivo studies confirmed its ability to adhere to skin, resist detachment during movement, and significantly accelerate wound healing with the regeneration of skin appendages such as hair follicles and blood vessels. Histological analysis supported these findings, showcasing effective tissue repair. Overall, this starch-based hydrogel offers a safe, sustainable, and highly effective platform for treating infected wounds and holds strong potential for broader applications in tissue engineering and regenerative medicine.

#### 6.1.7. Pectin-Based Hydrgels

Pectin is a natural polysaccharide that is water-soluble and non-toxic, mainly made up of α-D-galacturonic acid residues along with varying amounts of methoxy and ester groups depending on its origin [248]. When the degree of esterification is below 50%, referred to as low-methoxy pectin, it can create a three-dimensional gel network by interacting with divalent ions such as calcium (Ca^2+^), forming a structure often described as an “egg-box.” This distinctive configuration is especially useful for controlled drug delivery applications [249,250]. The characteristics of pectin-based hydrogels can be adjusted to suit different purposes—for instance, adding 2-thiobarbituric acid enhances flexibility and strengthens the structure [251], while the inclusion of zeolite improves oxygen permeability. Additionally, pectin can be chemically altered by introducing aldehyde groups, which allows the formation of injectable, biodegradable, and self-healing hydrogels when combined with poly(N-isopropylacrylamide) containing acylhydrazide functionalities [252]. There are some articles investigated the combination of pectin with other compounds like alginate or honey to make hydrogels for wound healing. One study focused on evaluating the potential application of a hydrogel composed of alginate and pectin in aqueous solution for creating innovative wound healing materials [253]. It also examined how the properties of this composite gel differ from those formed by each component separately. The interaction between sodium alginate and pectin within the combined gel matrix was analyzed, and its biocompatibility was confirmed. Results demonstrated that when alginate and pectin were mixed in equal proportions and dried, the resulting gel exhibited a notably more intricate structure compared to gels made from the individual polymers. Specifically, a fibrous network appeared at both micron and submicron levels, characterized by randomly oriented, densely packed fibers measuring 10–50 nm in width and 100–250 nm in length, which tended to aggregate into larger structures approximately 400 nm in size. Furthermore, the alginate–pectin composite maintained biocompatibility levels comparable to those observed in hydrogels formulated with either alginate or pectin individually. In another study, Giusto et al. [254], investigated the combination of pectin and honey to develop biomedical hydrogels for wound healing in rats. The results highlight a clear synergistic effect between the materials used to prepare the films, each contributing a variety of healing properties typically found separately in pharmaceutical products. The wound closure rate was notably quicker in the rats facing pectin-honey hydrogel, liquid honey, and pectin compounds, when compared to the control. With the pectin-honey hydrogel, the wound on rats showed a significantly accelerated healing process. Interestingly, the pectin demonstrated faster wound healing than the liquid honey; this difference was not statistically significant. These findings suggest that the combined materials have strong potential for enhancing wound healing, and specifically, the pectin-honey hydrogel formulation proves to be highly effective in promoting and speeding up tissue repair.

The polysaccharides discussed earlier remain the primary candidates for wound healing hydrogels. Typically, single-component hydrogels fall short of addressing all wound treatment demands. Therefore, composite hydrogels are designed to combine the strengths of multiple components, resulting in superior overall properties. Polysaccharides with unique features, like the antibacterial chitosan or the ion-cross-linkable alginate, attract significant attention. Ultimately, the choice of polysaccharide raw materials should be carefully tailored to the specific wound environment and the functional requirements of the dressing to ensure optimal healing outcomes.

### 6.2. Protein-Based Hydrogels

Protein-based hydrogels, derived from natural proteins like collagen, gelatin, silk fibroin, and fibrin, are highly valued in wound healing because of their biocompatibility, biodegradability, and inherent bioactivity. These hydrogels closely resemble the extracellular matrix, fostering cell adhesion, migration, and proliferation, which are crucial for tissue regeneration. Their high water content keeps the wound moist, supporting faster healing and less scarring. Also, many protein hydrogels can be designed to deliver therapeutic agents such as antibiotics or growth factors directly to the wound, enhancing healing. Their hemostatic properties help control bleeding, and their adjustable mechanical strength makes them suitable for both acute and chronic wounds.

#### 6.2.1. Gelatin-Based Hydrogels

Gelatin has emerged as a highly promising polymer scaffold for producing hydrogels that can form directly at the injury site, making it ideal for applications in wound healing and tissue regeneration. Its favorable characteristics—including inherent bioactivity, compatibility with living tissues [255], natural biodegradability, minimal immune response, and cost-effectiveness—have led to its approval by the US Food and Drug Administration (FDA) for use in medical fields [256]. The presence of various reactive groups in gelatin, such as primary amines, carboxyls, and hydroxyl groups, allows it to be chemically tailored through the attachment of diverse cross-linkers or therapeutic molecules, broadening its utility in regenerative medicine. Numerous physical and chemical crosslinking techniques have been established to develop gelatin-based hydrogels capable of gelation in situ. Consequently, gelatin hydrogels with bioactive properties have been designed as carriers for localized drug delivery, enabling controlled release profiles to enhance therapeutic outcomes [257]. A critical factor in this delivery system is the hydrogel’s ability to encapsulate drugs within its network by modulating pore size, charge properties, and incorporating bioconjugates [258]. Over the years, a wide range of therapeutic agents has been successfully embedded in gelatin hydrogels to promote effective wound treatment.

Desferrioxamine (DFO) is an iron-specific chelating agent that has received FDA approval for clinical management of conditions such as Mediterranean anemia. The molecule’s multiple hydroxyl groups effectively sequester free iron ions, preventing their involvement in the enzymatic hydroxylation and subsequent degradation of hypoxia-inducible factor-1 alpha (HIF-1α), thus stabilizing HIF-1α levels. This stabilization enhances the expression of angiogenic mediators including vascular endothelial growth factor (VEGF), as demonstrated in several experimental models. In a notable study, Chen et al. [259] engineered a photo-crosslinkable gelatin-based hydrogel system embedded with DFO to promote angiogenesis and facilitate wound healing in diabetic skin. Methacrylated gelatin (GelMA), synthesized to permit covalent crosslinking under light exposure, formed a three-dimensional matrix supporting cell growth and vascular network formation. A 15% (w/v) GelMA concentration was chosen based on its superior biomimetic properties, including effective cellular attachment, robust proliferation rates, and mechanical resilience suitable for in vivo tissue repair [260]. Controlled release assays confirmed sustained delivery of DFO from the hydrogel matrix for up to 72 h. In vitro studies with human umbilical vein endothelial cells (HUVECs) revealed enhanced cellular adhesion and the formation of capillary-like structures when cultured with the DFO-loaded GelMA hydrogel. Subsequent in vivo evaluation in diabetic murine models demonstrated accelerated wound closure, facilitated by enhanced epithelial migration and neovascularization, correlated with increased expression of HIF-1α and VEGF. These therapeutic effects of DFO via upregulation of HIF-1α and VEGF have also been reported in multiple studies [261]. Separately, Takei et al. [262] described a hydrophobically modified gelatin (HMG) hydrogel designed for the co-delivery of hydrophilic and hydrophobic agents. Gelatin’s amino acid composition includes both polar residues—such as hydroxyproline, serine, arginine, lysine, aspartic acid, and glutamic acid—and nonpolar residues—such as tyrosine, phenylalanine, tryptophan, leucine, and proline—enabling complex network formation through ionic and hydrophobic interactions. HMG was synthesized by grafting hydrophobic alkyl chains onto gelatin through Schiff base formation between the aldehyde groups of dodecanal and gelatin’s primary amines, followed by reductive amination to stabilize the modification. Gelatin type B, which carries a negative charge at physiological pH due to its isoelectric point (pI) of approximately 5.0, was used to encapsulate basic fibroblast growth factor (bFGF), a positively charged protein with a pI of around 9.6. The encapsulation was driven by electrostatic attraction between the oppositely charged molecules, enabling efficient loading of bFGF into the gelatin matrix. By adjusting the degree of substitution (DS) of alkyl groups, the balance of hydrophobicity and charge density within the hydrogel was modulated to optimize drug retention and release profiles. Current research efforts are increasingly directed towards the use of biomolecules, alongside conventional chemical drugs and growth factors, to enhance wound healing outcomes. Zheng et al. [263] developed injectable supramolecular gelatin hydrogels designed to deliver resveratrol and histatin-1 for the treatment of burn wounds. Their investigation focused on the dual role of resveratrol (Res) and histatin-1 (His-1) in modulating inflammatory responses and promoting vascular regeneration within damaged skin tissue. Resveratrol, a non-flavonoid polyphenolic antioxidant derived from grape leaves and skins, has been shown to activate the SIRT-1 signaling pathway, which regulates endothelial cell proliferation. Additionally, estrogen receptor-α has been implicated in enhancing endothelial cell-mediated wound repair, while AMP-activated protein kinase (AMPK) signaling contributes to vascularization and anti-aging effects. Histatin-1, a histidine-rich polypeptide synthesized by the parotid and submandibular salivary glands (which are two of the three major pairs of salivary glands in the human body. Their main role is to produce saliva, which helps with digestion, oral hygiene, and keeping the mouth moist), exhibits biological activities related to cell adhesion, including the facilitation of cell–substrate and cell–cell interactions. These properties have been linked to its potential in reinforcing epithelial barrier function and promoting neovascularization via activation of the Rab interactor 2 (RIN2)/Rab5/Rac1 signaling axis. To effectively administer these therapeutic molecules, the authors synthesized methacrylated gelatin (GelMA) and acryloyl-functionalized β-cyclodextrin (Ac-β-CD), which were combined to form a hydrogel network through lithium phenyl-2,4,6-trimethylbenzoylphosphinate (LAP)-mediated photopolymerization and host–guest interactions between cyclodextrin moieties and hydrophobic amino acid residues in GelMA. Resveratrol and histatin-1 were incorporated into this hydrogel matrix via the host–guest chemistry mechanism. In vitro assays with human umbilical vein endothelial cells (HUVECs) exposed to hydrogel extracts demonstrated significantly enhanced cellular migration and capillary-like tube formation, indicative of improved angiogenic potential. In vivo evaluation further confirmed the hydrogel’s therapeutic efficacy by revealing reduced expression of pro-inflammatory cytokines, including interleukin-6 (IL-6) and interleukin-1β (IL-1β), along with accelerated wound closure. Additionally, increased expression of angiogenesis-related markers such as CD31 and α-smooth muscle actin (α-SMA) supported the promotion of vascular remodeling during the healing process.

Collectively, these studies highlight the promise of gelatin-based hydrogels as effective delivery platforms for therapeutic agents that aim to enhance wound repair. The interplay between hydrogel porosity and surface charge optimizes drug loading and release kinetics, while simultaneously promoting host cell infiltration and interaction with the biomaterial. This synergy significantly enhances the bioactivity of encapsulated agents, underscoring the utility of gelatin hydrogels as multifunctional scaffolds for regenerative medicine applications.

#### 6.2.2. Collagen-Based Hydrogels

Collagen (Col) is a highly prevalent protein produced by the body that serves as the primary structural component in connective tissues, such as skin, tendons, and bones [264]. It is the most abundant protein found in the extracellular matrix [265]. Collagen plays various biological roles, including providing mechanical strength and structural integrity, mediating cell adhesion and motility, and modulating cellular proliferation and metabolic activities during tissue growth and repair. Currently, 28 distinct collagen types have been identified; among these, fibrillar collagen types I, III, and V primarily make up the skin’s collagen matrix [266]. Type I collagen is the most abundant, comprising approximately 70% of the total collagen content in the skin. Type III collagen is usually co-localized with type I and accounts for about 15% of collagen in the skin [267]. Other collagen forms, such as types IV, V, VI, and VII, constitute the remaining fraction [268]. Due to its biocompatibility, biodegradability, hemostatic capability, and ability to aid in tissue repair, collagen incorporation into wound dressings has been shown to significantly support the wound healing cascade. Collagen-based hydrogels, characterized by their porous structure and protofibrillar networks, enhance cellular infiltration and proliferation, facilitating tissue remodeling and accelerating wound recovery.

Recent studies classify hydrogel dressings for wound care into three distinct categories: (a) hydrogels comprised purely of collagen, (b) collagen-based composites combined with natural and/or synthetic polymers, and (c) collagen hydrogels embedded with bioactive compounds. The choice and integration of these components critically influence the hydrogel’s physicochemical characteristics, mechanical robustness, biocompatibility, and biological functionality, thereby affecting its overall therapeutic potential.

Collagen can serve as a carrier for therapeutic agents to enhance their biological effectiveness; however, it is often used in its pure form without incorporating bioactive substances. Considering that the addition of bioactive compounds may provoke adverse reactions [269], pure collagen remains a safer alternative. Numerous studies have shown that collagen-based hydrogels provide an excellent scaffold for wound dressings. For instance, Ge et al. isolated collagen from tilapia skin (PSC) and developed a hydrogel dressing containing 10 mg/mL of PSC [270]. NIH-3T3 fibroblasts cultured on these hydrogels for three days exhibited high biocompatibility and showed no cytotoxicity in MTT assays, indicating the suitability of PSC hydrogels for wound applications. Furthermore, in vivo experiments revealed that wounds treated with collagen hydrogels healed significantly faster than controls on days 14, 21, and 28 post-treatment. Similarly, Jridi et al. investigated the structural and rheological characteristics of collagen gels derived from cuttlefish skin and assessed their wound healing potential [271]. In rat models, treatment with this collagen gel for eight days resulted in accelerated wound closure, likely due to elevated hydroxyproline levels. These findings suggest that collagen gels facilitate extracellular matrix remodeling and expedite tissue repair, supporting their role as effective wound healing agents. Ying et al. developed an extracellular matrix (ECM)-mimicking hydrogel by covalently crosslinking collagen and hyaluronic acid (Col/HA) [268]. This composite hydrogel exhibited a significantly higher swelling ratio compared to hydrogels composed solely of collagen, attributed mainly to hyaluronic acid’s exceptional capacity for water retention. In vitro experiments demonstrated that human microvascular endothelial cells (HMECs) and fibroblasts (COS-7) cultured within the Col/HA hydrogel showed marked proliferative activity. In vivo assessment revealed that wounds treated with the Col/HA hydrogel developed the thickest granulation tissue layer, approximately 1300 µm, which was 300 µm thicker than in other test groups. The crosslinking of hyaluronic acid contributed to reduced hydrogel degradation while eliciting only a mild inflammatory response, confirming the hydrogel’s functional role in wound repair. In a separate study, Cao et al. engineered a hydrogel consisting of human-like collagen (HLC) and carboxymethylated chitosan (CCS) via an enzyme–chemical double-crosslinking strategy [272]. Mechanical testing showed that the HLC–CCS hydrogel achieved superior tensile strength (93.858 kPa) and breaking tensile modulus (112.068 kPa) compared to controls. The enhanced mechanical properties were attributed to HLC’s extended chain conformation, which enables the formation of a robust network upon crosslinking. The incorporation of CCS facilitated secondary crosslinking through EDC/NHS chemistry, producing stable covalent bonds that further reinforced the hydrogel’s tensile characteristics. Functionally, HLC–CCS hydrogels promoted wound healing by aiding hemostasis, enhancing macrophage-to-fibroblast transdifferentiation, and improving mechanical resilience. Immunohistochemical analyses revealed increased secretion of growth factors such as VEGF and CD31 by cells within the hydrogel matrix. Additionally, Lei et al. reported the synthesis of a hydrogel dressing composed of human-like collagen, carbonylated chitosan (CCS), and hyaluronic acid, further exploring multifunctional biomaterial formulations for wound care applications [273].

Incorporation of diverse bioactive compounds into wound dressings is a common strategy to amplify their therapeutic efficacy. Recently, the integration of functionalized nanoparticles has emerged as an innovative approach to reinforce and stabilize collagen-based matrices, thereby improving their structural integrity and biological performance.

Zhao et al. engineered a collagen-based hydrogel incorporating chitosan and silver ions (COCAg) [274], which demonstrated potent antimicrobial efficacy. This hydrogel exhibited injectability and self-healing capabilities, attributed to dynamic Schiff base chemistry. To enhance the hydrogel’s inherent low stability, a double-network structure was established through a combination of photocrosslinking and Schiff base reactions. In vivo evaluations showed that the COCAg-treated wounds harbored the fewest bacterial colonies. By day 14, complete wound closure was observed in the COCAg group, whereas wounds treated with collagen-chitosan (COC) hydrogels or gauze remained unhealed, underscoring the COCAg hydrogel’s potential for managing infected wounds. Collagen, a naturally hydrophilic bioactive polymer, can be chemically modified to deliver hydrophobic drugs effectively in wound care applications. Olivetti et al. synthesized collagen hydrogels grafted with dodecenylsuccinic anhydride (DDSA) to facilitate the delivery of the hydrophobic anti-inflammatory agent simvastatin [275]. The DDSA modification preserved the hydrogel’s fibrous and porous architecture while increasing its hydrophobicity, enabling efficient drug loading. Although DDSA grafting reduced cellular adhesion and proliferation within the scaffolds, the hydrogels maintained favorable cytocompatibility and supported cell spreading. Simvastatin incorporation suppressed pro-inflammatory cytokines associated with the M1 macrophage phenotype, leveraging its combined antibacterial and anti-inflammatory effects to promote skin wound healing. Additional research has highlighted metformin’s ability to polarize macrophages toward the M2 phenotype, thereby attenuating inflammation in type 2 diabetic murine models [276].

#### 6.2.3. Fibrin-Based Hydrogels

Fibrin is an endogenous protein polymer generated through the enzymatic conversion of fibrinogen, primarily mediated by thrombin during the coagulation cascade. This biopolymer forms a three-dimensional fibrous matrix that exhibits notable biocompatibility and is subject to enzymatic degradation under physiological conditions. Its structural features contribute to favorable mechanical behavior, such as viscoelasticity and the ability to undergo strain-stiffening under mechanical load. Functionally, fibrin supports critical cellular activities including adhesion, proliferation, and directed migration. Moreover, it plays a multifaceted role in physiological processes such as blood clot stabilization, modulation of inflammatory responses, and facilitation of tissue repair [277].

Fibrin-based hydrogels are typically synthesized by combining fibrinogen and thrombin, often supplemented with coagulation factor XIIIa to enhance cross-linking and generate a mechanically robust, insoluble fibrin matrix. Experimental optimization has demonstrated that the relative concentrations of fibrinogen, thrombin, and factor XIIIa significantly impact the biomechanical characteristics of the hydrogel, which in turn modulate cell behaviors such as migration, differentiation, spreading, and proliferation via mechanotransductive signaling pathways. For example, one investigation reported that reduced fibrinogen and thrombin levels were associated with enhanced cellular proliferation. Thrombin is frequently dissolved in a calcium chloride (CaCl_2_) solution to ensure an adequate supply of calcium ions necessary for the activation of factor XIIIa, which further stabilizes the fibrin network [278]. Sodium chloride (NaCl) concentration is another key factor influencing the physicochemical profile of fibrin hydrogels. Gels formulated with physiological salt (PS) levels at 145 mM and high salt (HS) levels at 250 mM exhibit distinct degradation kinetics. Notably, the in vitro degradation time of HS gels is approximately threefold longer than that of PS gels. In vivo, PS gels fully degrade within one week following subcutaneous implantation, whereas HS gels remain structurally intact, highlighting their potential as sustained-release platforms [279].

To prevent premature degradation, tranexamic acid—a non-toxic antifibrinolytic agent—has been used to maintain scaffold integrity during the early stages of cell growth. In addition to showing high biocompatibility and supporting efficient cell attachment, fibrin hydrogels also have a consistent porous structure with high porosity levels. This microstructure increases surface area, promoting cell–matrix interactions, stimulating angiogenesis, and enabling efficient exchange of nutrients and waste products. Empirical studies have shown that an optimal pore size of approximately 20–124 μm is especially effective in supporting fibroblast growth and migration, which are essential for effective wound healing.

Moreover, fibrin hydrogels exhibit pronounced hydrophilicity, allowing them to absorb substantial amounts of water and swell accordingly [280]. This characteristic enables rapid uptake of wound exudates and sustains a moist local environment, both of which are critical for promoting tissue repair and providing a protective barrier at the wound site [281]. Fibrin is also recognized for its intrinsic anti-inflammatory and antioxidant effects, contributing to a regenerative microenvironment conducive to tissue restoration. For instance, in a study by Pereira et al. [277], application of fibrin hydrogels to full-thickness dermal wounds resulted in reduced inflammatory responses, evidenced by lower levels of pro-inflammatory cytokines and elevated interleukin-10 (IL-10). Enhanced proliferation and migration of skin endothelial cells, fibroblasts, and keratinocytes were also observed, collectively accelerating the wound healing process.

From a mechanical standpoint, fibrin hydrogels demonstrate viscoelastic properties, behaving as both solids and fluids under varying conditions, which supports their use as dynamic scaffolding materials for regenerative medicine applications [282]. These properties are particularly advantageous in guiding neural development. For example, fibrin hydrogels fabricated with lower fibrinogen concentrations have been shown to more effectively promote the extension of dorsal root ganglion neurites in murine models, compared to those with higher concentrations [283]. Furthermore, the injectable nature of fibrin hydrogels allows them to conform closely to irregular tissue geometries, addressing limitations associated with prefabricated solid scaffolds that lack tissue conformity.

#### 6.2.4. Silk Fibroin-Based Hydrogels

Silk protein hydrogels composed exclusively of silk-derived biomaterials, without the inclusion of exogenous bioactive agents such as cells, growth factors, or small molecules, represent a class of pure protein-based scaffolds. In the study conducted by Chouhan et al. [284], a mixture of silk protein solutions (3% *w*/*v*) derived from both mulberry and tussah silkworms underwent spontaneous self-assembly at physiological temperatures, resulting in hydrogel formation within 15–20 min. This thermo-responsive gelation behavior enables in situ hydrogel formation under body temperature conditions. Compared to traditional collagen-based hydrogels, this silk protein formulation demonstrated superior therapeutic efficacy in treating full-thickness skin injuries, notably reducing local inflammatory responses and accelerating epithelial regeneration, ultimately promoting functional recovery in third-degree burn wounds. In a separate investigation, riboflavin (RF) and ammonium persulfate (APS) were employed to initiate photo-crosslinking of silk fibroin, forming a gel upon exposure to white light via photo-oxidation of tyrosine residues [285]. When applied to second-degree burns in rodent models, this gel supported scar-free wound healing. However, the photo-crosslinking process required approximately 20 min, rendering it less suitable for rapid in situ gelation during clinical applications. To address this limitation, a more rapid gelation method was developed using glycidyl methacrylate (GMA)-modified silk fibroin. This approach employed lithium phenyl (2,4,6-trimethylbenzoyl) phosphate (LAP) as a photoinitiator, with ultraviolet (UV) light at 380 nm enabling gelation within just 10 s [286]. This method offers a highly efficient strategy for achieving fast, in situ gel formation, making it particularly advantageous for clinical scenarios requiring immediate application and solidification of hydrogel dressings.

Therefore, this rapid photo-crosslinking system is well-suited for both fast in situ gelation and digital light processing (DLP)-based 3D bioprinting applications. In our study, both the in situ-formed hydrogels and the DLP-printed silk fibroin scaffolds demonstrated strong regenerative potential in skin wound models. Notably, the 3D-printed scaffolds, owing to their predefined architecture, provided enhanced structural support and facilitated deep cellular infiltration, making them particularly effective for treating large-area skin defects. Furthermore, single-cell transcriptomic analysis revealed that the photo-crosslinked silk fibroin hydrogel established a regenerative microenvironment conducive to efficient skin repair, indicating its ability to modulate cell behavior at the molecular level and promote coordinated tissue regeneration.

It is well established that promoting vascularization during skin defect repair plays a pivotal role in alleviating local hypoxia, enhancing nutrient transport, and facilitating the recruitment and migration of regenerating cells to the wound site. To this end, researchers have explored strategies to functionalize silk protein hydrogels with pro-angiogenic agents to accelerate and improve the skin regeneration process.

For instance, Wang et al. [287] incorporated the angiogenic peptide Nap–Phe–Phe–Ser–Val–Val–Tyr–Gly–Leu–Arg—an analog of vascular endothelial growth factor (VEGF)—into silk protein aqueous solutions, enabling the formation of self-assembling, stable hydrogels. Their in vivo experiments demonstrated that these peptide-functionalized hydrogels could stimulate neovascularization as early as 3 days following subcutaneous implantation, significantly expediting functional skin regeneration. In a related study, Liu et al. [288] developed silk protein nanofiber hydrogels via thermal dialysis and utilized them as delivery vehicles for the plant-derived compound asiaticoside. These asiaticoside-loaded hydrogels exhibited both anti-inflammatory and angiogenic properties, contributing to effective skin defect repair. Wu et al. [289] advanced this approach by embedding mesenchymal stem cells (MSCs) within desferrioxamine-loaded silk protein hydrogels and applying them to skin flap models. The inclusion of MSCs synergistically enhanced neovascularization and reduced inflammatory responses, thereby improving the therapeutic efficacy of the desferrioxamine-loaded hydrogels.

Given the critical role of the immune microenvironment in wound healing outcomes, researchers have focused on modulating local immune responses through the integration of immunoregulatory agents. Lee et al. [290] incorporated epigallocatechin gallate (EGCG), a potent antioxidant, into silk protein hydrogels. This modification provided the hydrogels with the ability to scavenge reactive oxygen species (ROS), thereby reducing oxidative stress at the wound site and promoting tissue regeneration. Similarly, Tang et al. [291] used polydopamine-reduced graphene oxide to enhance ROS scavenging in silk hydrogels. Additionally, because of its electrical conductivity, this composite material facilitated signal transmission that stimulated cellular proliferation and growth. Furthermore, Bhar et al. [292] embedded aloe vera gel extract into silk protein hydrogels, improving their immunomodulatory function. Their findings indicated significant downregulation of pro-inflammatory cytokines such as interleukin-1β (IL-1β) and tumor necrosis factor-α (TNF-α), along with upregulation of anti-inflammatory markers including interleukin-10 (IL-10) and transforming growth factor-β (TGF-β), thus fostering a regenerative microenvironment.

Collectively, these studies underscore the inherent immunomodulatory and regenerative properties of silk protein hydrogels, which can be further tailored through the incorporation of functional molecules such as peptides, drugs, cells, and antioxidants. Compared to composite silk-based scaffolds, pure silk protein hydrogels offer distinct advantages in terms of cost-efficiency, batch-to-batch consistency, and scalability, rendering them particularly attractive for clinical translation and large-scale therapeutic applications in skin tissue engineering.

#### 6.2.5. Peptide-Based Hydrogels

Peptide-based hydrogels have emerged as promising biomaterials for the treatment of chronic wounds due to their tunable properties that can be tailored to meet specific therapeutic demands [293]. Given their natural abundance in the human body, peptides exhibit inherent biological functionality, including selective bioactivity and biocompatibility. The unique structural and functional attributes of peptide-based hydrogels render them particularly suitable for chronic wound care applications, allowing for customized therapeutic design. Notably, several peptides derived from amphibian skin secretions—such as OA-GL12, OA-GL21, RL-QN15, and Ot-WHP—have demonstrated the ability to accelerate wound healing processes. These effects have been observed in both acute and chronic wounds, including those associated with diabetic conditions [294].

The current advantages of peptide-based hydrogels include their low production cost, straightforward synthesis, and high structural diversity, making them adaptable for a range of biomedical uses. In vivo safety evaluations have indicated that, upon degradation, these hydrogels exhibit minimal toxicity and high biocompatibility [295,296]. Moreover, the degradation kinetics of peptide hydrogels can be finely regulated through the design of peptide monomers and the controlled application of proteolytic enzymes such as trypsin and α-chymotrypsin, which cleave amide bonds [297]. These features support the development of biodegradable and pH-responsive peptide hydrogels, which are highly advantageous for applications in drug delivery and wound dressing systems. In recent years, the concept of self-assembly in biological systems has garnered significant interest due to its ability to produce functional supramolecular architectures from discrete macromolecular building blocks. Among these, peptide-based hydrogels constitute a prominent class of biomaterials, notable for their inherent self-assembling properties [298,299]. These hydrogels are typically composed of short peptides or amino acids and are capable of entrapping large amounts of water or biological fluids within their three-dimensional networks. Under physiological conditions, they undergo nanoscale organization to form hydrogel structures [300,301].

The assembly and gelation of peptide hydrogels are primarily driven by non-covalent interactions, including hydrogen bonding, hydrophobic interactions, π–π stacking among aromatic residues, and electrostatic forces [302,303]. Common approaches to initiate gelation include thermal modulation—such as heating followed by cooling—and alterations in pH. Furthermore, the addition of salts to peptide solutions, especially under alkaline conditions, is frequently employed to enhance molecular self-assembly and promote gel formation. These self-assembled peptide hydrogels often display sensitivity to external stimuli, such as changes in pH, temperature, mechanical stress, ionic strength, and the composition of biological fluids. However, this stimuli-responsive behavior is not a universal trait and is highly dependent on the specific amino acid sequence and the physicochemical environment governing self-assembly. Of particular interest are formulations that demonstrate thixotropic properties—defined by their ability to undergo reversible sol–gel transitions upon application and removal of shear stress. This thixotropy is not a general characteristic of all peptide hydrogels but is limited to systems with distinct structural configurations and intermolecular interaction profiles that enable such dynamic mechanical responsiveness.

Over the past decade, self-assembled peptide hydrogels have emerged as promising alternatives to conventional scaffolding materials in regenerative medicine. These hydrogels spontaneously organize into nanofibrous networks that emulate the architecture of native extracellular matrices (ECM), thereby providing a structural and biochemical framework conducive to tissue regeneration. Their capacity to support angiogenesis underscores their utility in soft tissue repair, particularly in applications involving vascular and dermal reconstruction [304]. At the nanoscale, the assembled peptide fibers facilitate the formation of direct interfaces between damaged tissues and the ECM, enhancing cell migration and integration at the wound site.

A prototypical example is the ionic self-complementary peptide RADA16-I, which has been extensively studied for its regenerative potential in neural tissue. It has demonstrated efficacy in promoting axonal regeneration, synapse formation, and wound healing, including restoration of impaired optical pathways [179]. RADA16-I assembles into ECM-mimetic nanofibrous scaffolds that provide a supportive microenvironment for in vivo cellular proliferation. Importantly, its enzymatic degradation produces L-amino acids that are endogenously metabolized, mitigating the immunological risks and contamination issues commonly associated with animal-derived materials like collagen [305]. Nevertheless, a key limitation of RADA16-I is its acidic pH, which may adversely affect cell viability in three-dimensional culture systems.

To address this issue, Silva et al. developed a composite hydrogel incorporating the IKVAV (Ile-Lys-Val-Ala-Val) sequence (derived from laminin) and the RGD motif (from fibronectin), both functional at physiological pH to enhance compatibility and regenerative outcomes. Complementary work has introduced peptide-based hydrogels formed from L-lysine-derived peptides that self-assemble into helical nanofibers [306]. In vivo studies using rat models with partial-thickness burn wounds demonstrated that these hydrogels maintained optimal hydration levels, resulting in accelerated wound healing.

In comparative studies, two peptide hydrogels were evaluated alongside Mepitel^®^, a clinically approved silicone-coated polyamide mesh dressing. Initial results revealed that the peptide hydrogels enhanced autolytic debridement and stimulated both re-epithelialization and dermal tissue regeneration, without the requirement for additional therapeutic agents. These ultrashort peptide-based materials effectively addressed key limitations of existing wound care systems. Furthermore, their functionality can be expanded by incorporating bioactive molecules such as antimicrobial agents, pharmaceuticals, cytokines, and growth factors, thereby enhancing their therapeutic potential in advanced wound management strategies [307].

Peptide-based hydrogels have demonstrated considerable promise in skin regeneration, offering immunologically inert scaffolds suitable for treating a range of skin injuries, from superficial abrasions to full-thickness burns. Their biocompatibility and tunable bioactivity make them particularly well-suited for managing chronic wounds. Recent preclinical and clinical investigations have identified Connexin43 (Cx43) as a therapeutic target to enhance cutaneous healing, particularly through its role in re-epithelialization. One notable study examined ACT1, a 25-amino acid peptide that mimics the C-terminal domain of Cx43, in the treatment of chronic, non-healing neuropathic diabetic foot ulcers [308]. Administered in a hydroxyethyl cellulose hydrogel to patients with ulcers persisting for at least four weeks, ACT1 significantly reduced wound dimensions, promoted epithelial closure, and enabled full epidermal restoration without eliciting immunogenic or adverse effects. In a synergistic therapeutic approach, the combination of adipose-derived stem cells and Exendin-4—a glucagon-like peptide-1 receptor agonist—was shown to significantly accelerate wound healing. This combination enhanced the migration, proliferation, and invasion of human endothelial cells and keratinocytes, resulting in faster wound closure and improved skin regeneration outcomes.

The versatility of peptide-based hydrogels as multifunctional platforms in regenerative medicine is further supported by research demonstrating their potential in chronic wound management, especially in diabetic ulcers. For example, Balaji et al. reported that peptide nanofibers (NFs) could facilitate the formation of an in situ tissue-engineered provisional matrix (ISTEPM), which significantly modulated the diabetic wound microenvironment [309]. This matrix supported granulation tissue development, promoted neovascularization, and suppressed inflammation, collectively contributing to improved wound closure. The formation of ISTEPM was also shown to overcome diabetes-induced impairments in vascular infiltration and early angiogenesis. In another study, Carrejo et al. evaluated multidomain peptide (MDP) hydrogels in diabetic mouse models with full-thickness dermal wounds and observed complete wound closure within 14 days [310]. Treated wounds exhibited advanced tissue remodeling, including dense granulation tissue, enhanced vascularization, nerve fiber integration, and hair follicle neogenesis—features indicative of high-quality tissue regeneration. Complementary work by Nidadavolu et al. introduced a self-assembling filamentous hydrogel formulation of valsartan (val-filament) for diabetic wound treatment in Zucker Diabetic Fatty (ZDF) rats [311]. The val-filament hydrogel enabled sustained drug release and acted as a structural scaffold, achieving full wound closure in all treated animals by day 23, in contrast to only one closure in the control group. Post-inflammatory phase analyses indicated improved healing outcomes, increased hair follicle density, enhanced mitochondrial bioenergetics, and modulation of cellular adhesion and Smad/TGF-β signaling pathways. Gao et al. further demonstrated the regenerative efficacy of peptide hydrogels through the incorporation of substance P, which augmented angiogenesis and collagen deposition in diabetic wound models [312]. Similarly, Kim et al. found that treatment with self-assembling peptides (SAP) in combination with substance P (R+SP and R+R−SP) significantly reduced wound size, predominantly due to enhanced tissue regeneration. After three weeks, wounds treated with substance P exhibited advanced healing, likely mediated by the recruitment of mesenchymal stem cells (MSCs), which play a critical role in early wound repair [313].

Additional support for the efficacy of peptide hydrogels comes from studies on Lys–Lys–(Ser–Leu)_6_–Lys–Lys-based multidomain peptide nanofiber systems. These hydrogels were shown to stimulate granulation tissue formation, re-epithelialization, angiogenesis, and neural regeneration in diabetic wounds [314]. Furthermore, Mandla et al. demonstrated that treatment with Q-peptides not only facilitated wound closure in diabetic mouse and rat models but also triggered systemic tissue regeneration, suggesting therapeutic potential beyond localized wound repair [315].

Finally, a study by Wan et al. assessed a novel hydrogel derived from scorpion peptides for ulcer therapy and found that it significantly accelerated healing compared to epidermal growth factor (EGF), particularly during the second week of treatment and continuing through to complete resolution [316]. The superior healing performance was attributed to its enhanced anti-inflammatory and antimicrobial activity—key therapeutic features in the management of chronic diabetic wounds.

## 7. Beyond the Benefits: A Closer Look at the Risks and Challenges of Natural Hydrogels

While natural hydrogels are widely recognized for their biocompatibility, biodegradability, and ability to mimic the ECM, a truly comprehensive and clinically relevant review must also address the risks and limitations of their use. These challenges, often overlooked in the literature, are crucial for preclinical studies and successful clinical translation [317]. A more thorough discussion of these issues can help bridge the gap between research and real-world application, especially for clinicians and regulatory agencies. To improve clarity and accessibility, the main risks associated with natural hydrogels are summarized in Table 7, with a focus on their clinical implications.

## 8. Conclusions and Future Perspectives

This review begins by outlining the intricate stages of wound healing and the multifactorial elements that influence this dynamic physiological process, which requires the coordinated interplay of cells, signaling molecules, and extracellular matrix (ECM) components. Given the limitations of conventional wound care strategies, significant efforts have focused on the development of advanced biomaterials, among which natural hydrogels derived from biological macromolecules such as polysaccharides, proteins, and peptides have emerged as promising candidates. These hydrogels create a moist wound environment, closely mimic the native ECM, and can be engineered for controlled delivery of therapeutic agents, thereby making them highly suitable for treating a broad spectrum of wounds, ranging from superficial injuries to chronic, non-healing ulcers.

Polysaccharide-based hydrogels provide excellent biocompatibility, biodegradability, and inherent bioactivity, including antimicrobial and hemostatic properties. Protein-based hydrogels—composed of collagen, gelatin, and silk fibroin—are especially valued for their structural resemblance to skin ECM, supporting cellular adhesion and migration. Peptide-based hydrogels, due to their modular design and intrinsic biofunctionality, allow precise control over their assembly, degradation, and biological activity. Collectively, these natural hydrogel systems exhibit essential properties such as stimuli responsiveness, tunable porosity, and drug-loading capacity that meet the complex requirements of wound healing. Emerging preclinical and clinical evidence demonstrates that these hydrogels not only accelerate re-epithelialization and granulation tissue formation but also enhance angiogenesis, modulate immune responses, and in some cases, promote neural regeneration and hair follicle neogenesis. Peptide-based hydrogels, in particular, are gaining recognition for their ability to deliver bioactive sequences and form intelligent systems that respond dynamically to physiological cues within the wound microenvironment.

Despite their biological advantages and promising laboratory and early clinical outcomes, the widespread clinical adoption of natural polymer-based hydrogels—especially those derived from chitosan, alginate, and gelatin—remains limited. This limitation is primarily due to regulatory complexities, cost-effectiveness concerns, and comparative performance challenges relative to established commercial products such as Integra^®^ and Apligraf^®^ [318]. Regulatory hurdles pose a significant barrier, as natural hydrogels may be classified variably as medical devices, biologics, or combination products depending on their composition and therapeutic application. Unlike Integra^®^ and Apligraf^®^, which have undergone rigorous FDA premarket approval (PMA) processes supported by extensive clinical validation and long-term safety data, natural hydrogels often suffer from a lack of standardized formulations, inconsistent mechanical performance, and limited long-term safety and scalability data, complicating regulatory approval [133].

From an economic perspective, although raw materials for natural hydrogels are generally cost-effective, the overall cost–benefit ratio is negatively impacted by factors such as shorter shelf life, storage constraints, lower mechanical robustness necessitating more frequent dressing changes, and limited reimbursement support. In contrast, clinically validated products like Integra^®^—a bilayer matrix composed of bovine collagen and glycosaminoglycan with a silicone outer layer—and Apligraf^®^—a living bilayered cell therapy approved for diabetic foot ulcers and venous leg ulcers—offer superior clinical reliability, consistent and predictable healing outcomes, and are well-integrated into reimbursement frameworks. Consequently, despite their significant biological potential, natural hydrogels have not yet displaced engineered skin substitutes in clinical practice, largely due to ongoing challenges related to regulatory pathways, mechanical consistency, and economic viability.

Looking forward, the future of wound care with natural hydrogels lies in the development of next-generation multifunctional systems. These advanced platforms are expected to integrate smart features such as on-demand drug release, bioresponsive degradation, and real-time wound monitoring through embedded biosensors. The incorporation of stem cells, exosomes, growth factors, and gene-editing tools into hydrogel matrices further expands their therapeutic potential, opening new avenues for regenerative wound therapy. Additionally, biofabrication technologies including 3D bioprinting and microfluidics promise the customization of hydrogel scaffolds to match patient-specific wound geometries and healing requirements. Despite these promising advancements, challenges including scalability, regulatory compliance, long-term biocompatibility, and cost-effectiveness remain critical obstacles for clinical translation. Addressing these challenges will require multidisciplinary collaboration among materials scientists, clinicians, bioengineers, and regulatory experts.

In summary, natural hydrogels represent a transformative approach in wound healing with the potential to redefine therapeutic standards for both acute and chronic wounds. Continued innovation and strategic development are poised to deliver highly effective, personalized therapies that not only facilitate tissue repair but also restore its full function and integrity.

## Figures and Tables

**Table 1:** Overview of Wound Healing Phases and Their Timeframes.

Phase	Description	Timeframe
Hemostasis	Blood clotting begins immediately to stop bleeding.	Seconds to hours
Inflammation	White blood cells clean the wound and fight infection.	1–14 days
Proliferation	New tissue and blood vessels form; epithelial cells cover the wound.	4 days to several weeks
Remodeling	Collagen is reorganized, and the wound strengthens and closes.	Weeks to months

**Table 2:** Overview of Intrinsic and Extrinsic Factors Influencing Wound Healing.

Category	Factor	Impact on Healing
**Intrinsic**	Age	Slower cell regeneration and reduced immune response
	Nutrition	Deficiencies (protein, vitamins A and C, zinc) impair tissue repair
	Chronic Diseases	Conditions like diabetes or cardiovascular disease delay healing
	Immune Status	Immunocompromised individuals experience slower healing
	Genetics	Certain genetic conditions affect collagen production or inflammation
	Hormonal Imbalance	Stress hormones (e.g., cortisol) can suppress healing
**Extrinsic**	Infection	Extends inflammation and causes further tissue damage
	Oxygenation	Poor blood flow or hypoxia limits cell activity and vessel formation
	Mechanical Stress	Movement or pressure disrupts tissue repair
	Temperature and Moisture	Optimal warmth/moisture aids healing; extremes hinder it
	Medications	Drugs like steroids and chemotherapy impair the healing process
	Smoking and Alcohol	Reduce circulation and weaken immune response

**Table 3:** Key differences between acute and chronic wounds.

Feature	Acute Wounds	Chronic Wounds
Onset	Sudden (e.g., trauma, surgery, burns)	Gradual or persistent (e.g., ulcers, pressure injuries)
Healing timeline	Predictable, typically within 2–4 weeks	Prolonged, often >6 weeks; may stagnate or worsen
Healing phases	Progresses through normal stages: hemostasis → remodeling	Disrupted or stalled in inflammation or proliferation
Etiology	Usually an isolated injury	Multifactorial: poor perfusion, infection, comorbidities
Infection risk	Lower, short-term exposure	Higher, often biofilm-associated and resistant
Tissue environment	Healthy surrounding tissue	Often ischemic, necrotic, or fibrotic
Clinical management	Standard wound care protocols	Requires advanced, multidisciplinary interventions

**Table 4:** Classification and Evaluation of Conventional Wound Treatment Modalities.

Type of Dressing	Source/Material/Methods	Advantages	Disadvantages
**Dry gauze dressings**	Cotton or synthetic fibers	- Inexpensive - Widely available - Easy to apply	- Allows the wound to dry out [60] - Adheres to the wound bed - Painful removal - Limited antimicrobial protection [61]
**Non-adherent films**	Polymer-based (PE, silicone)	- Minimizes trauma during dressing changes - Transparent for wound monitoring	- Poor exudate absorption - May require secondary dressing - Biologically inert
**Hydrocolloids**	Gel-forming agents (CMC)	- Maintains a moist environment [62] - Promotes autolytic debridement [54]	- Not suitable for infected wounds - May cause odor or maceration - Limited antimicrobial action
**Basic antimicrobial ointments**	Topical agents (antibiotics)	- Reduces surface bacterial load - Easy to apply	- Rapid, uncontrolled release - Requires frequent reapplication - Risk of resistance development
**Foam dressings**	Polyurethane foam	- Absorbs moderate exudate [63] - Provides cushioning - Maintains moisture	- May dry out the wound if not properly managed - Limited bioactivity
**Alginate dressings**	Seaweed-derived polysaccharides	- Highly absorbent [64] - Promotes moist healing [65] - Biocompatible	- Not ideal for dry wounds - Requires secondary dressing - Not antimicrobial properties
**Impregnated gauze**	Gauze with additives (petrolatum, iodine)	- Reduces adherence - Delivers basic antimicrobial or healing agents	- Limited moisture retention - May still cause trauma during removal
**Natural substances and Poultices**	Honey, resin salve, poultices, and herbal remedies	- Natural accessible sources - Ability to fight infection or inhibit microbial growth [66] - Promote wound healing through granulation or anti-inflammatory effects [67]	- May vary in potency, sterility, or cause allergic reactions [68] - Possible interaction with other treatments - Sticky texture can be inconvenient
**Debridement techniques**	Surgical, mechanical or enzymatic method	- Promote wound healing - Clean wound environment for regeneration [69] - Reduce infection risk [70]	- May cause pain and discomfort [71] - Invasive procedure - Risk of bleeding or damaging healthy tissue
**Compression and Offloading**	Mechanical	- Reduces edema and promotes healing - Standard method for diabetic foot and venous ulcers [72]	- Requires proper assessment and skilled application - Not suitable for all patients [73]
**Vacuum/NPWT**	Mechanical suction via sealed dressing system	- Removes exudate - Stimulates granulation tissue - Promotes angiogenesis and wound contraction - Effective for chronic and complex wounds [32]	- High costs for equipment - Specialized training for safe application [74] - May cause pain and discomfort - Not suitable for all wound types
**Oxygen therapy**	Delivers oxygen via a hyperbaric chamber	- Improves oxygen delivery to hypoxic tissue - Stimulates granulation tissue formation [75] - Enhances immune response and collagen production - Effective in ischemic wounds and diabetic foot ulcers	- Expensive and resource-intensive - Requires trained personnel and strict protocols [76] - Not suitable for all patients
**Surgical closure**	Primary/Secondary/Tertiary closure Skin grafting, flap surgery	- Simple with reduced rejection risk - Enhance healing process - Suitable for complex wounds [77]	- Surgical risks (bleeding, infection) [78] - Risk of flap necrosis - May require revision
**Pain management**	Pharmacological	- Improves patient comfort and quality of life - Reduces inflammation and post-surgical care [79]	- Can masks signs of infection - Interfere with wound healing process [80] - Risks of side effects
**Holistic Systems**	Herbs, diet, traditional remedies, and meditation techniques	- Natural herbs and food to stimulate healing [81] - Deep cultural integration [82] - Integrates body–mind healing	- Lack of standardization - Variable preparation method - Difficult to validate scientifically [83]

**Table 5:** Examples of Commercially Available and Clinically Studied Hydrogel Dressings.

Product	Hydrogel Composition	Indication	Key Outcomes/Features
**DermaSyn™, Purilon^®^, Intrasite™**	CMC/alginate, amorphous	Ulcers, burns	Faster closure, pain relief [148]
**AQUACEL^®^ Foam**	Hydrofiber/foam hybrid	Chronic/acute wounds	Moisture control, fewer change [149]
**ChitoCare gel**	Chitosan	DFU, chronic ulcers	Granulation, faster healing [150]
**EHO-85 gel**	Plant extract-based	Mixed etiology	area reduction [151]
**AmnioGraft^®^, NuCel**	Decellularized amnion	Burns/Chronic	Rapid epithelization, low scarring [152]
**DermiSphere hDRT**	Collagen hydrogel	Deep/full-thickness	FDA-cleared, tissue integration [153]
**HydroClean^®^ (polyacrylate-based)**	Polyacrylate hydrogel	Leg ulcers	Superior debridement/granulation [154]

**Table 6:** Classification of natural hydrogels.

Type	Hydrogel	Source	Key Benefits in Wound Healing	Mechanical Properties	Degradation Rate	Refs.
Polysaccharide-Based	**Alginate**	Brown seaweed	Moisture retention, hemostasis, easy gelation	Variable stiffness; e.g., alginate β-sheet hybrid hydrogels span ~0.6–205 kPa	Up to ~20% dissolution within 7 days in buffer	[178]
	**Chitosan**	Crustacean shells	Antimicrobial, hemostatic, promotes healing	Composite gels (e.g., with silk fibroin/HA) show elastic modulus ~1.8 kPa; up to ~15 kPa	Degradation slowed by composite integration; specific rates depend on formulation	[179,180]
	**Hyaluronic Acid**	Connective tissue ECM	Cell migration, anti-inflammatory, hydration	Enhanced via double cross-linking, offering improved strength and tunability.	Can be tailored through chemical modification and cross-link density adjustments.	[181]
	**Cellulose**	Bacterial (*G. xylinus*)	Maintains a moist environment	LiCl/DMAc–derived cellulose hydrogels showed high compressive and tensile strength	Volume shrinkage during regeneration correlated with mechanical robustness	[182]
	**Dextran**	Bacterial polysaccharide	Biocompatible scaffold	Enhanced when multivalent crosslinkers (NPGDA, TMPTA, PETA) are used; IPN systems exhibit significantly greater stiffness.	Higher crosslink density (e.g., via increased dextran-MA) leads to reduced swelling and controlled degradation.	[183]
	**Starch**	Plant-derived	Absorbs exudate, maintains moist environment	~1096 kPa compressive strength; >85% recovery after 20 load–unload cycles	~81–86% weight loss after 180 days under soil biodegradation conditions	[184]
	**Pectin**	Plant cell walls	Moisture retention, biocompatible	Young’s modulus ranged from 6 to 100 kPa; higher pectin concentration increases stiffness	Faster degradation in PBS compared to DMEM and fibroblast cultures; medium influences rate	[185]
Protein-Based	**Gelatin**	Animal collagen	Cell adhesion, biodegradable	Young’s modulus of 7.16 kPa for hydrogels crosslinked with H_2_O_2_ and HRP for 60 min	Modulated by H_2_O_2_ exposure time; suitable for muscle cell sheet applications	[186]
	**Collagen**	Animal connective tissue	Tissue regeneration, cellular support	Twisting and crosslinking enhanced mechanical strength and toughness significantly.	Degradation slowed with increased crosslinking and densification of collagen fibers.	[187]
	**Fibrin**	Blood plasma protein	Hemostasis, natural tissue scaffold	Enhanced with increasing dextran-MA concentration—yielding reduced swelling and smaller mesh sizes	Tunable: higher Dex-MA content suggests slower degradation via denser network structure	[188]
	**Silk Fibroin**	Silkworm cocoons	Mechanical strength, cell support	Tailorable mechanical properties suitable for skin tissue engineering applications.	Slower degradation with higher β-sheet content; influenced by processing conditions.	[189]
Peptide-Based	**Self-Assembling Peptides**	Natural short peptides	Promote angiogenesis, endothelial growth, tissue regeneration	Stiffness (elastic modulus) ranged from 0.6 to 205 kPa, modulated by sequence, concentration, and buffer.	Up to 20% dissolution after 7 days in buffer; enzymatic and bacterial degradation were slower.	[178]

**Table 7:** Key Risk Factors and Clinical Implications of Natural Hydrogels.

Risk Factor	Description	Clinical Implication
**Immunological Reactions**	Natural polymers—especially those derived from animal sources like collagen or chitosan—may contain residual proteins, endotoxins, or other impurities that trigger immune responses.	These reactions can lead to localized inflammation, delayed wound healing, or even rejection of the hydrogel material.
**Source Variability**	The composition of natural polymers varies significantly based on origin, harvesting method, and processing. For instance, polysaccharides from marine or plant sources may differ in purity, molecular weight, or bioactivity.	Such variability can compromise reproducibility, alter mechanical properties, and lead to inconsistent therapeutic performance across batches.
**Sterilization Challenges**	Natural hydrogels are highly sensitive to conventional sterilization techniques like heat, gamma radiation, or ethylene oxide. These methods can degrade the polymer structure or alter its bioactivity.	Ineffective or damaging sterilization may reduce shelf stability, compromise mechanical integrity, or result in unsafe clinical products.
**Degradation Products**	As natural hydrogels break down, they may release acidic or immunogenic byproducts, depending on the polymer type and degradation pathway. Without proper characterization, these can negatively interact with the surrounding tissue.	This necessitates comprehensive toxicological profiling and clearance studies to ensure that degradation does not interfere with healing or trigger adverse responses.
